# Harnessing the Power of Polyphenols: A New Frontier in Disease Prevention and Therapy

**DOI:** 10.3390/ph17060692

**Published:** 2024-05-27

**Authors:** Mohamed El Oirdi

**Affiliations:** 1 Department of Life Sciences, College of Science, King Faisal University, Al Ahsa 31982, Saudi Arabia; meloirdi@kfu.edu.sa; 2 Department of Basic Sciences, Preparatory Year, King Faisal University, Al Ahsa 31982, Saudi Arabia

**Keywords:** polyphenols, flavonoids, non-flavonoids, action mechanisms, therapeutic uses

## Abstract

There are a wide variety of phytochemicals collectively known as polyphenols. Their structural diversity results in a broad range of characteristics and biological effects. Polyphenols can be found in a variety of foods and drinks, including fruits, cereals, tea, and coffee. Studies both in vitro and in vivo, as well as clinical trials, have shown that they possess potent antioxidant activities, numerous therapeutic effects, and health advantages. Dietary polyphenols have demonstrated the potential to prevent many health problems, including obesity, atherosclerosis, high blood sugar, diabetes, hypertension, cancer, and neurological diseases. In this paper, the protective effects of polyphenols and the mechanisms behind them are investigated in detail, citing the most recent available literature. This review aims to provide a comprehensive overview of the current knowledge on the role of polyphenols in preventing and managing chronic diseases. The cited publications are derived from in vitro, in vivo, and human-based studies and clinical trials. A more complete understanding of these naturally occurring metabolites will pave the way for the development of novel polyphenol-rich diet and drug development programs. This, in turn, provides further evidence of their health benefits.

## 1. Introduction

Polyphenols, potent bioactive compounds present in numerous plant sources, have garnered considerable interest in scientific investigations and nutritional studies due to their noteworthy health-promoting properties. These compounds are abundant in a wide range of plant-based foods, from the green leaves of tea plants (*Camellia sinensis* L.) and the strong beans of coffee (*Coffea arabica* L.) to wholesome soybeans and juicy grapes (*Vitis vinifera*). Studies have demonstrated that polyphenols encompass a diverse array of molecules, each characterized by its unique intricate structure. This group includes flavonoids, phenolic acids, stilbenes, and lignans, highlighting the diversity within polyphenols [1,2,3]. 

The structural complexity of polyphenols, marked by their phenolic rings and hydroxyl substituents, underpins their categorization into flavonoids and non-flavonoids, each group boasting its own subclasses and health implications. The primary structural difference between flavonoids and non-flavonoids is that flavonoids have two phenol rings, while non-flavonoids only have one ring. Additionally, a central pyran ring containing oxygen connects the two phenol rings in flavonoids. Flavonoids can be divided into six subclasses: flavonols, flavones, isoflavones, flavanones, anthocyanidins, and flavanols [3,4].

The diversity in the structures of these molecules plays a critical role, supporting a wide array of biological functions that range from antioxidant to anti-microbial effects. These functions are crucial for reducing oxidative stress, decreasing chronic inflammation, and slowing the advancement of age-related diseases, such as cardiovascular diseases, neurodegenerative conditions, and cancer [5,6,7].

The chemopreventive and therapeutic capabilities of dietary polyphenols in combating such diseases have been extensively substantiated, demonstrating their effectiveness in enhancing cardiovascular and metabolic well-being, decreasing the likelihood of diabetes and obesity, and presenting encouraging prospects in addressing Alzheimer’s disease by impeding amyloid-beta aggregation [8,9,10]. 

Furthermore, the interaction of polyphenols with the gut microbiota emerges as a key mechanism through which these compounds exert their health benefits. This highlights the importance of their bioavailability and bioaccessibility in optimizing their positive effects on human health [3,9,11,12].

### 1.1. Bioavailability

There is no direct relationship between the amount of polyphenols in food and their ease of absorption or utilization by the body. Polyphenols are absorbed into the bloodstream through the intestinal mucosa when ingested orally, then transported to the desired tissues. Several studies validate the growing interest in nutraceuticals [13]. One’s dietary habits dramatically impact the regulation of multiple metabolic activities. Food serves not only as sustenance for the body’s metabolic functions but also contains bioactive compounds that contribute to health benefits, including antioxidants, vitamins, polyunsaturated fatty acids, and fiber [14]. Thus, consuming a nutritious diet and its various components might enhance an individual’s well-being, reduce their likelihood of acquiring specific diseases, and overall improve their quality of life [14,15].

Understanding how polyphenols are absorbed and distributed throughout the body is essential for assessing their potential impact on biological activity in living organisms and their overall significance in preventing various diseases linked to oxidative stress [16,17].

Research on absorption is challenging due to the intricate molecular composition of polyphenol-rich foods and other factors, including the level of polymerization and conjugation with other chemicals and phenols. Most polyphenols exist in food as esters, glycosides, or polymers and cannot be absorbed in these forms [18]. After intake, polyphenols are recognized as xenobiotics, leading to their comparatively poor bioavailability compared to micro- and macronutrients [19].

Recent studies on the bioabsorption of polyphenols suggest that their bioavailability is limited when consumed in relatively high concentrations [20]. The primary challenge to their pharmacological utilization is their low bioavailability, associated with the interactions of polyphenols at various stages of digestion, absorption, and distribution that modify their molecular structure, particularly their interactions with food, digestive enzymes, and transporters in the intestine and blood proteins [21,22].

Very small amounts of consumed polyphenols are absorbed in the small intestine. Enterocytes and hepatocytes can hydrolyze and biotransform simpler polyphenolic substances after absorption. Hydrophilic conjugated metabolites such as methyl, glucuronide, and sulfate derivatives are then rapidly released into the bloodstream, transported to various organs, or eliminated in urine [23,24,25]. The potential formation of new compounds during the metabolism of each phenolic molecule complicates tracing individual compounds in the body [26]. Metabolic activities typically transform phenolic antioxidants into entirely new compounds, making it nearly impossible to identify the parent phenol [27]. Phase I metabolism in enterocytes involves oxidation, reduction, or hydrolysis processes, introducing or exposing functional groups like hydroxyl groups [28].

Following absorption in the small intestine, the metabolites of polyphenols are delivered to the liver via the portal vein and reach systemic circulation. Glucuronides and dimethyl glucuronides are the primary metabolites detected in the portal vein [29]. Most metabolites are eliminated by the kidneys, but it has also been discovered that these polar conjugates can pass the hepatocyte membrane and undergo further modification in other cell types [22,30].

The vast majority of consumed polyphenols ultimately end up in the colon, covering both unabsorbed compounds from the small intestine and those that are absorbed and processed in either the liver or small intestine [31]. They are either transported back into the colon lumen through membrane transporters or bile. In the second stage of metabolism, the breakdown of polyphenols is accompanied by the production of numerous new metabolites by the abundant microbiota found in the colon [31,32].

Bacterial enzymes can catalyze a wide variety of reactions, including rapid deconjugation. The enzymatic degradation of flavonoids in the colon results in the production of numerous new metabolites [33,34]. The gut microbiota’s enzymes break down the flavonoid scaffold into phenolic acids and other simpler compounds. The extent of flavonoid metabolites’ presence in the intestines is yet to be fully understood. Therefore, understanding how gut microbes affect flavonoid distribution and bioactivity in food is crucial [35,36].

To improve the absorption and health benefits of dietary polyphenols, future research and reviews must explore other methods to optimize processing conditions based on specific food compositions [37,38]. Developing and using food-grade bio-based nanocarriers to encapsulate, store, and distribute polyphenolic chemicals is a recently proposed solution to the limited bioavailability of these compounds. The application of bio-based nano-complexes extracted from polyphenol food-grade sources in nutraceuticals provides an intellectually stimulating opportunity, with the potential to alter the advancement and utilization of nutraceuticals by merging nano-delivery techniques with the advantageous properties of polyphenols [23,39,40].

### 1.2. The Aim of this Study

The abundant presence of these metabolites in plant-based diets, coupled with compelling evidence linking their consumption to a reduced risk of cancer, diabetes, obesity, and cardiovascular diseases, has undoubtedly propelled them to the forefront of scientific discussions. A high-polyphenol-rich diet has been associated with a decreased risk of several chronic diseases related to aging, as supported by both epidemiological and clinical research [3,4,38]. The primary aim of composing this review was to highlight certain important, but underappreciated, aspects of polyphenols and their role in maintaining human health. Specifically, this review is intended to shed light on the function of polyphenols in assessing the merits and demerits of several investigations.

While conducting this literature review, the author sought out the most impactful and up-to-date research on chemical and biological properties by searching in vitro, in vivo, and clinical studies. The emphasis was on gathering information from 305 relevant and recent papers on polyphenols. The studies centered on diseases such as cancer, diabetes, microbial infections, obesity, arthritis, immunological conditions, neurological disorders.

### 1.3. Data Sources

A thorough literature search for English-language reports was conducted, utilizing the latest versions of databases such as Web of Science, Google Scholar, Scopus, and PubMed. The search terms included “polyphenols, flavonoids, non-flavonoids in diabetes, obesity, arthritis, cancer, microbial, cardiovascular, immunomodulatory, and neurological action”, as well as various subclasses of polyphenols. The sections on beneficial pharmacological properties, possible mechanisms of flavonoids and non-flavonoids, and preclinical and clinical trials primarily focus on the latest scientific research to the greatest extent possible.

**Figure 1:**
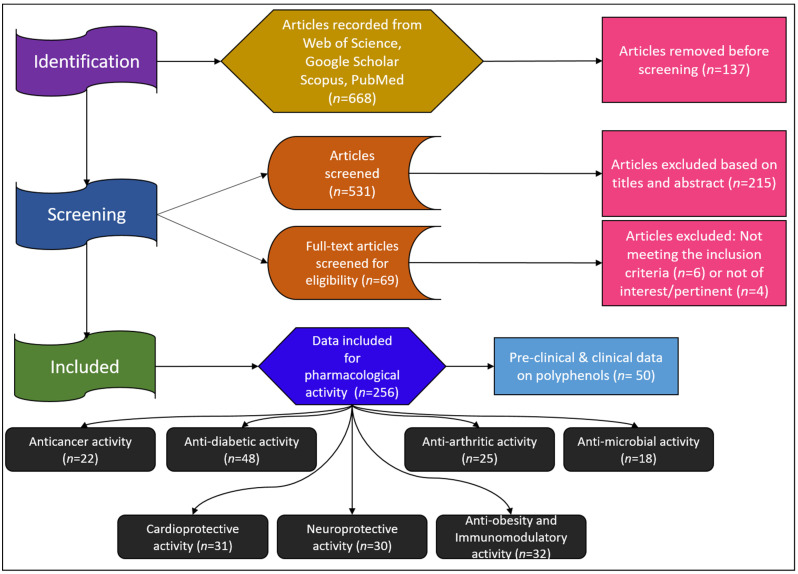
From search to selection: PRISMA flowchart of review methodology.

The study selection process, according to PRISMA guidelines, is depicted in Figure 1. A total of 668 records were identified from the database search. After removing 137 duplicate articles, 531 studies were screened, and 215 were excluded based on the title and/or abstract. The full texts of the eligible studies (*n* = 69) were read, and 12 studies were excluded for not meeting the inclusion criteria (*n* = 6) or being irrelevant/not pertinent (*n* = 4). At the end of the selection process, 256 papers were included regarding the pharmacological activity of polyphenols. The relevant references were exported to the Zotero reference manager. The study selection and characteristics were determined using the guidelines of the Preferred Reporting Items for Systematic Reviews and Meta-Analyses (PRISMA) [41].

## 2. Recent Studies and Emerging Trends on Polyphenols

### 2.1. Anticancer Activity

**Figure 2:**
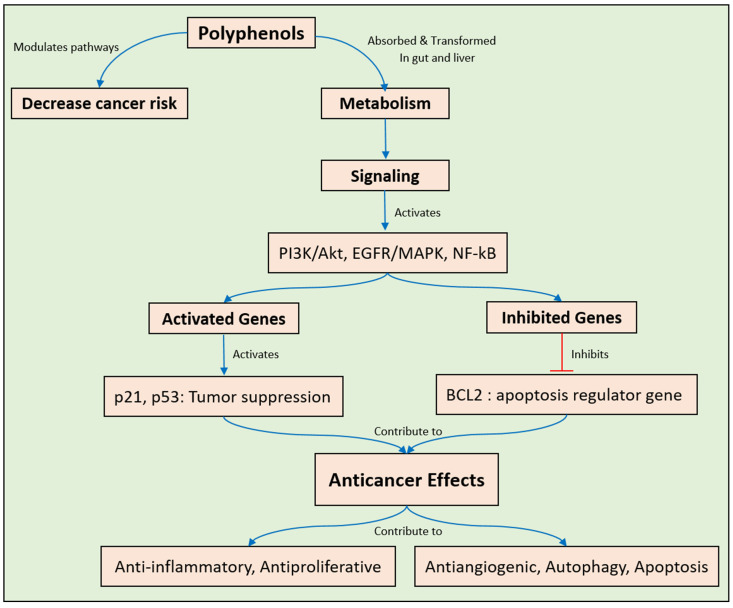
The protective effects and action mechanisms of dietary polyphenols against cancer.

Epidemiological research indicates that cancer pathologies are linked to urban lifestyles, dietary habits, and environmental factors [42,43]. Individuals are prone to experience a variety of harmful side effects connected with present cancer therapies such as chemotherapy, radiotherapy, and immunotherapy [44]. The natural world has often served as a reservoir for novel bioactive compounds in the pursuit of effective chemotherapy agents, with plant polyphenols emerging as the most recognized beneficial dietary constituents. Owing to their structurally diverse nature, they manifest a broad spectrum of biological properties [43]. Evidence from various observational, experimental, and clinical inquiries has highlighted a significant relationship between regular polyphenol consumption and a reduced cancer risk [43]. Through the modulation of multiple cellular signaling pathways via interactions with various target proteins, polyphenols can elicit their anticancer effects [45]. Nonetheless, challenges such as high metabolic susceptibility, limited cell-membrane permeability, minimal systemic exposure, physiological variations, and oxidative stress pose major obstacles to the effective therapeutic use of polyphenolic compounds. These substances can influence the processes of carcinogenesis through diverse mechanisms [46]. Polyphenols feature anti-inflammatory, antiproliferative, anti-angiogenic, autophagic, and apoptotic functions [43,44,47]. They are able to aim for numerous cellular processes, such as gene expression, cell cycle proliferation, cellular migration, and progression, to demonstrate their anticancer effects. In most cases, the antioxidant actions of polyphenolic compounds underlie their cytoprotective and anticancer characteristics [48]. By modulating important signaling pathways (PI3K/Akt, EGFR/MAPK, NF-κB) (Figure 2), polyphenols can (i) neutralize reactive oxygen species (ROS) and other free radicals; (ii) reduce DNA mutations and damage; (iii) inhibit the cell cycle; (iv) induce apoptosis; and (v) downregulate cell proliferation. Furthermore, polyphenols may exert anticancer effects through various pathways, such as the cell death receptor pathway, mitochondria-mediated apoptosis via ROS generation, and the perforin-granzyme apoptotic route [43,49]. Moreover, phenolic substances can decrease tumor expression through the p53 pathway while also regulating metabolism and cell formation (Figure 2). Beyond repairing DNA damage in cancer cells, they can inhibit DNA replication and RNA transcription [50].

One of the purposes of this review is to appraise the potential of polyphenols as supplements in cancer prevention by studying recent clinical and preclinical trials that concentrate on their capability to lessen the detrimental impacts of anticancer treatments. One study conducted by researchers highlighted that ellagic acid, a substance existing in diverse fruits and nuts, exerts a potent anticancer effect on breast cancer cells, particularly MCF-7 cells. Upon exposure to gamma radiation, ellagic acid was observed to enhance cell apoptosis, diminish cell colony formation, and induce a higher rate of cells entering the apoptotic phase, a programmed cell death stage [51]. Notably, the experiment illustrated that ellagic acid heightens the sensitivity of MCF-7 cells to radiation, exhibiting a synergistic impact that surpasses the effects of ellagic acid or radiation by themselves. Moreover, in normal mouse cells, ellagic acid exhibited a protective rather than harmful influence, underscoring its potential for use in the selective targeting of cancer cells.

In another independent research endeavor, scientists explored the influence of oleuropein, a compound obtained from olives, on ovarian cancer cells. Their research exposed that oleuropein intervention elevated the activity of distinct proteins that obstruct tumor growth while lessening those that foster it, leading to diminished resistance to the chemotherapy drug cisplatin, decreased cell proliferation, and increased cell death [52]. Furthermore, scientists have paid attention to hydroxytyrosol, a breakdown derivative of oleuropein, for its anti-inflammatory features and its capability to lessen oxidative stress, notably in decreasing kidney harm brought about by cisplatin in mice [53].

Shifting the focus towards honey, this natural sweetener transcends its conventional use as a delicacy. Its intricate blend of compounds, varying based on the floral sources visited by bees and their geographical location, encompasses elements such as coumaric acids and flavonoids. These components not only contribute to health benefits but also hold promise in cancer prevention according to recent studies [54]. For instance, investigations on rodents have revealed honey’s potential in inhibiting the progression of breast cancer and enhancing immune function, potentially serving as a natural support for cancer therapy [55,56].

Moreover, honey exhibits the potential to mitigate oral mucositis, a distressing consequence of chemotherapy, through enhancing the body’s immune response. Clinical trials have underscored the benefits experienced by patients with oral mucositis from interventions involving honey, including a honey–coffee combination, thereby highlighting the therapeutic prospects of honey [57]. Side effects in the throat, such as xerostomia, were less severe in radiation patients who consumed thyme honey [58]. Additionally, mice treated with cisplatin showed reduced liver and kidney damage when given manuka and talh honey, as reported in [59]. It is suggested that the anti-inflammatory, anti-apoptotic, and free-radical-scavenging properties of honey contribute to its organ-protective effects. These findings highlight honey’s potential for protective benefits and usefulness.

**Figure 3:**
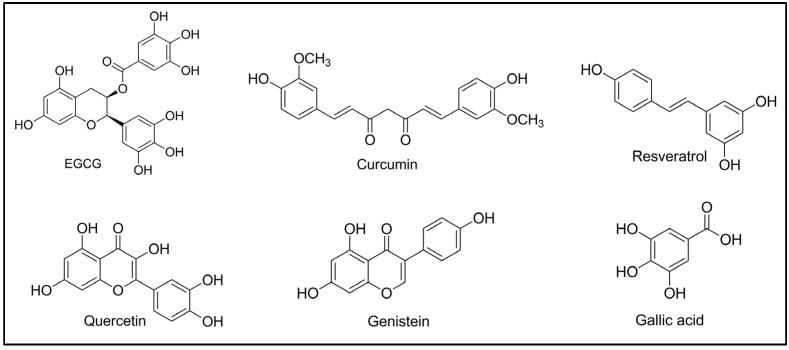
Chemical structures of EGCG, curcumin, resveratrol, quercetin, genistein, and gallic acid.

A clinical trial is currently underway to investigate the effects of a polyphenol-rich aerosol on reducing the side effects related to radiation therapy [60]. In this trial, ten patients undergoing radiotherapy for head-and-neck cancer will receive an aerosol containing hyaluronic acid, *Cetraria islandica*, vitamin B3, and plant extracts rich in polyphenols for one month. Furthermore, another clinical trial is exploring the use of a commercially available beverage called Nutridrink, which is fortified with a blend of plant extracts high in polyphenolic compounds, for patients recovering from gastrointestinal tumors [60]. Recent patents have introduced new formulations and approaches using quercetin (Figure 3) and its analogs to address radiation-induced bystander effects [61]. Moreover, one clinical trial is recruiting participants to examine the Mediterranean Intervention for Neurodegenerative Delay (MIND) diet’s ability to alleviate the neurotoxic side effects of chemotherapy in a subgroup of breast cancer patients [62]. There is preliminary evidence that the anti-inflammatory ingredients in the MIND diet, including omega-3 polyunsaturated fatty acids (PUFAs), carotenoids, B vitamins, and polyphenols, could help reduce the cognitive side effects of cancer treatments. Another clinical trial suggests that dietary supplementation with polyphenols may lessen some of the adverse effects of radiation therapy in breast cancer patients [63,64]. From the above available data, it is clear that polyphenols generated from plants provide a plethora of health benefits, including anticarcinogenic action. The pharmacological evidence for polyphenol’s anticancer effects is strong, and it is based on multiple mechanisms. These molecules may be used in the future for designing novel anticancer supplements for the prevention and treatment of carcinogenesis.

### 2.2. Anti-Diabetic Activity

**Figure 4:**
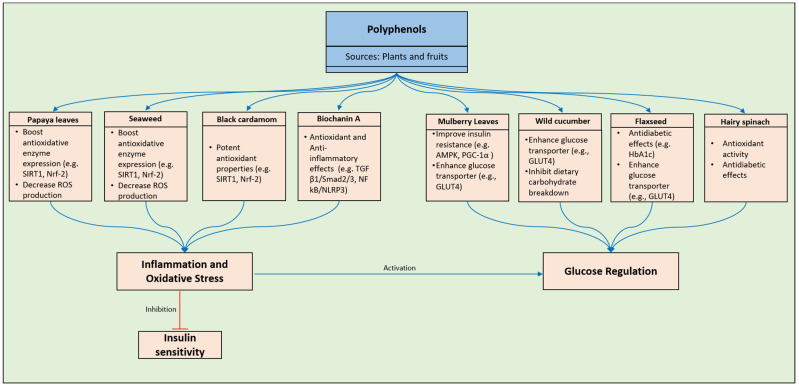
The protective effects and action mechanisms of dietary polyphenols against diabetes.

With a global prevalence exceeding 400 million individuals, type 2 diabetes (T2D) is a multifaceted metabolic disorder characterized by persistent inflammatory processes. The development of insulin resistance, coupled with insufficient insulin release and reduced anabolic activity in target tissues, disrupts metabolic pathways, leading to the onset of a potentially fatal chronic metabolic condition [65,66]. Throughout history, the utilization of seaweed and tropical papaya as traditional remedies has been prevalent. Studies involving animals have demonstrated the potential efficacy of papaya leaves and seaweed in the treatment of diabetes. These natural substances not only protect β-cells from diabetes-related harm but also contribute to reductions in fasting plasma glucose levels and A1C levels, the enhancement of antioxidative enzyme expression, and the mitigation of ROS production [65,66]. Analogous to various fruits and vegetables, seaweed and papaya contain a rich array of antioxidants, including vitamin A, C, and E complexes, as well as polysaccharides, phenolic compounds, essential fatty acids, saponins, fucoidans, and phlorotannins. The utilization of flavonoids in isolation may not completely alleviate all manifestations of diabetes [65]. One research endeavor revealed that diabetic mice subjected to treatment with the natural flavonoid linarin exhibited decreased levels of inflammation and oxidative stress [67]. Linarin appears to mitigate oxidative stress and inflammation in a model of hepatocyte damage induced by high levels of glucose and high levels of palmitic acid, as well as in a rat model of T2D, by inhibiting aldo-keto reductase (AKR)1B, an NADP(H)-oxidoreductase [68]. The category of flavonoids, which are phenolic compounds, encompasses isorhamnetin, a methylated derivative of quercetin [69]. Through the inhibition of aldose reductase, isorhamnetin displays promising potential as a therapeutic approach for diabetes [70]. Upon reviewing the notable impacts of isorhamnetin on glucose levels, oxidative status, inflammation, and lipid metabolism in both in vitro and in vivo settings, it is postulated that isorhamnetin could serve as a beneficial agent for managing diabetes [71]. A commonly used dietary spice, *Amomum tsao-ko* Crevost & Lemarie (black cardamom) is abundant in flavonoids [72]. Findings from various animal and laboratory studies indicate that methanol extracts of *A. tsao-ko* exhibit strong antioxidant and anti-diabetic properties. Further research is necessary to validate the specific mechanisms involved in the treatment of diabetes and oxidative stress using *A. tsao-ko*. The remarkable antioxidant and anti-inflammatory characteristics of apigenin have attracted significant attention due to its relevance in numerous physiological processes [73]. Its actions are mediated through the neutralization of superoxide, singlet oxygen, and hydroxyl radicals, as well as the enhancement of PPARγ signaling function and the inhibition of CD38 [74,75,76]. In vitro and in vivo investigations have illustrated that the flavonoid licochalcone A, derived from licorice, may possess both preventive and therapeutic properties towards diabetic nephropathy [77,78]. Within the bark of the *Myrica esculenta* plant, a flavone named myricitrin has been detected. Experimental findings have revealed its significant capability to reduce blood glucose levels in animal models of T2D, including rats and mice [72]. The activation of the IRS-1/PI3K/Akt/GLUT4 signaling pathway via myricitrin, as evidenced in both in vitro and in vivo studies, enhances glucose uptake by skeletal muscles. Investigations conducted in laboratory settings as well as on living organisms have shown that myricitrin can mitigate oxidative stress by stimulating nuclear factor erythroid 2-related factor 2 (Nrf-2) and counteracting oxidative radicals effectively (Figure 4) [72,79].

Among the numerous health benefits of isoflavones, biochanin A is widely recognized for its anti-inflammatory, antihyperlipidemic, antioxidant, and anticancer attributes by suppressing TGF-β1 and PAR-2 gene expression in kidney tissues of STZ-induced diabetic rats [80,81,82]. In addition to its antioxidant properties, biochanin A appears to exert an anti-diabetic influence. Animal models of T2D have exhibited enhanced insulin sensitivity [83], reduced glucose tolerance [80], and diminished glycohemoglobin A1C production. In a T2D rat model, biochanin A effectively maintains blood glucose levels within the normal range [84]. One meta-analysis identified a correlation between heightened levels of transforming growth factor-β (TGF-β) and an elevated risk of nephropathy [85]. Renal epithelial, endothelial, and podocyte cells serve as the primary sites of expression for all four subtypes of protease-activated receptors (PAR1-4) [86]. The inhibition of PAR-2 has been found to enhance autophagy while preventing inflammation and fibrosis [86]. Contrary to expectations, biochanin A mitigates diabetic nephropathy by downregulating the expression of the TGF-β1 and PAR-2 genes [65,81,82].

Formononetin, an isoflavone classified under the phytoestrogen category, induces cell death through the intrinsic apoptosis pathway, resulting in the permeabilization of the outer mitochondrial membrane [87,88]. Among the diverse biological effects of formononetin are its antioxidant properties [89] and its anti-diabetic actions, as demonstrated in laboratory and in vivo studies. Sirtuin 1 (SIRT1), a histone deacetylase that shields cells from ROS, plays a crucial role in hepatic lipid metabolism by upregulating AMP-activated protein kinase, which subsequently inhibits hepatic lipogenic pathways and enhances fatty acid oxidation [65,90]. One mechanism through which formononetin reduces blood glucose levels involves increasing SIRT1 expression in pancreatic cells (Figure 4) [80]. Reducing cytoplasmic lipid accumulation is a primary function of sirtuin-induced fatty acid oxidation. Despite the promotion of fatty acid oxidation, muscle glucose metabolism may be disrupted [91]. Formononetin, known for its potent apoptotic-inducing properties, is also believed to have such characteristics [65,87]. Multiple action pathways of formononetin have been identified [89,92,93]. Its potential as an adjunctive therapy for diabetic neuropathy and nephropathy, as well as its enhancement of various aspects of metabolic syndrome, including diabetes, makes formononetin a subject necessitating further investigation and analyses. There exist two distinct pathways through which fisetin demonstrates its anti-diabetic effects [94]. The initial mechanism by which fisetin inhibits gluconeogenesis involves reducing the cytosolic NADH/NAD(+) potential redox and hindering the transportation of pyruvate into the mitochondria [94]. Additionally, fisetin decreases blood glucose levels by impeding glycogen breakdown [95,96]. One promising therapeutic approach involves the utilization of fisetin, which exhibits the potential to complement other anti-diabetic medications. The flavonoid myricetin is prevalent in various teas, plants, and fruits [97]. Recent discoveries have unveiled the mechanisms of action of myricetin in diabetes, such as DPP4 inhibition [98], GLP-1 inactivation, or acting as a GLP-1 receptor agonist [99]. Notably, myricetin appears to normalize gut flora in T2D-afflicted mice [100]. Anthocyanins, which are polyphenolic compounds classified under the flavonoid group, regulate digestive enzymes (α-amylase and α-glucosidase), GLUT-4, GLP-1, glucose-6-phosphatase (G6Pase), phosphoenolpyruvate carboxykinase (PEPCK), and PPARγ. By modulating insulin production and resistance, these compounds exert control over blood glucose levels [101]. Pancreatic β-cells benefit from crucial protective mechanisms, including their antioxidant and anti-inflammatory properties [102]. The capacity of anthocyanins to regulate multiple enzyme types is deemed highly unlikely. Researchers are advocating for further exploration of the therapeutic potential of anthocyanins in treating diabetes, emphasizing the necessity for standardized and quantified studies to establish universal conclusions regarding their efficacy [65,101]. Extracts from *Delonix regia* have been found to display antioxidant, hypolipidemic, and hypoglycemic effects [103]. Notably, these extracts showcase anti-diabetic properties comparable to those of the renowned anti-diabetic drug glibenclamide, which lowers blood glucose levels by enhancing pancreatic insulin secretion. However, the components of *Delonix regia* extracts responsible for these antioxidant, hypoglycemic, and potentially harmful effects remain unidentified. Reports suggest that mulberry (*Morus alba* L.) leaves offer advantages for skeletal muscle function [104]. Mulberry leaves are a widely used and efficacious traditional Chinese remedy for managing blood sugar levels. In diabetic mice, the flavonoids present in mulberry leaves appear to ameliorate insulin resistance in skeletal muscles and enhance mitochondrial function through the AMPK-PGC-1α signaling pathway [105,106]. These leaves contain flavonoids that induce hypoglycemic effects by enhancing antioxidase activity and inhibiting the TGF-β1 pathway [65,106]. While the findings of studies on mulberry leaves are intriguing, they require validation through additional randomized controlled trials. In non-obese T2D Goto-Kakizaki rats, EGCG has been identified as having a dual function, potentially acting as a regulator of autophagy as well as an inhibitor of inflammation-associated gene expression in both peripheral leukocytes and adipose tissue [65,107]. The prooxidant activity of EGCG is attributed to its instability and autoxidation processes [108]. Procyanidins are composed of oligomers of catechin and epicatechin molecules. Some research suggests that plants rich in procyanidins may have a beneficial impact on reducing hyperglycemia and T2D [109]. Another study delves into the intricate details of catechins’ involvement in diabetes management, encompassing their structural features, classification, and underlying mechanisms [110].

Phytoestrogen lignan and polyphenols derived from *Linum usitatissimum* (commonly known as flax or linseed) have exhibited anti-diabetic properties in rats with streptozotocin-induced diabetes [111]. The consistent administration of flaxseed extract leads to an improvement in HbA1c levels and blood glucose levels, while also notably reducing total cholesterol, high-density lipoprotein (HDL), low-density lipoprotein (LDL), and triglyceride levels in diabetic rats.

A recent investigation explored the potential health advantages of *Byttneria pilosa* (hairy spinach), a flowering plant traditionally used to remedy conditions like boils and scabies. The antioxidant and anti-diabetic characteristics of the methanol extract of *B. pilosa* leaves (MEBP) were evaluated both in vitro and in vivo. The MEBP exhibited substantial effects in terms of anti-diabetic and antioxidant properties. A molecular docking analysis unveiled favorable interactions between beta-sitosterol and specific targets. Nevertheless, despite these encouraging outcomes, further inquiry is imperative to comprehensively grasp the mechanisms and therapeutic potential of MEBP [112].

Moreover, another documented research endeavor scrutinized the impact of polyphenols on exacerbating hyperglycemia in *Cucumis dipsaceus* fruits (wild cucumber). The antioxidant properties of these polyphenols were found to impede the breakdown of dietary carbohydrates and mitigate the effects of T2D mellitus. Additionally, a link was established between the anti-diabetic attributes of these phenolic compounds and their bioaccessibility, suggesting that they show potential for use in diabetes prevention and postponement [113].

Therefore, the inclusion of polyphenols in one’s diet may aid in managing blood glucose levels and potentially delaying the onset of diabetes. The conclusions drawn from this review underscore the necessity of natural elements for sustaining health. Furthermore, as demonstrated earlier, polyphenols have the capacity to ameliorate insulin resistance and high blood sugar levels in individuals with diabetes (Figure 4). Prior to their introduction as anti-diabetic remedies, these phenolic compounds should undergo supplementary cytotoxicity assessments, clinical trials, and preclinical investigations.

### 2.3. Anti-Arthritic Activity

Numerous inflammatory chronic diseases have been the focus of polyphenol research because of their potential antioxidant, anti-inflammatory, and immunomodulatory effects. This portion of the present review aims to describe how these substances can affect the inflammatory pathways that are characteristic of the most common types of arthritis, such as rheumatoid arthritis (RA) and osteoarthritis (OA).

**Figure 5:**
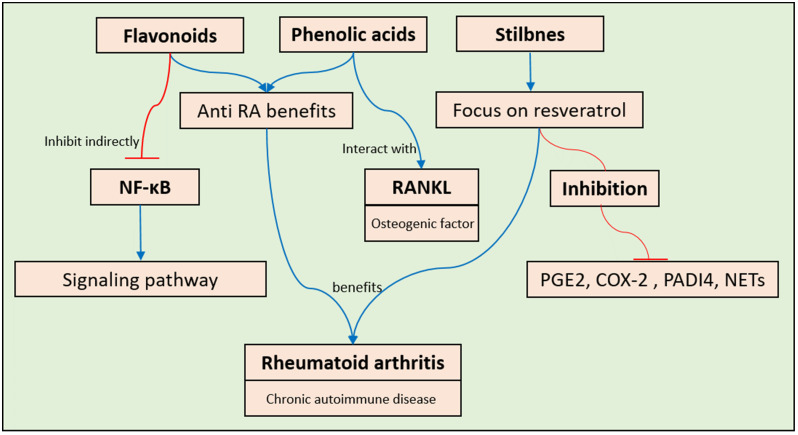
The protective effects and action mechanisms of dietary polyphenols against arthritis.

RA is a chronic autoimmune inflammatory disease that specifically affects the joints. It has the potential to cause damage to both cartilage and bone [114]. This condition is distinguished by inflammation of the synovial tissue, swelling, the formation of autoantibodies, and the loss of cartilage and bone. Flavonoids exhibit many pathways that have anti-rheumatoid-arthritis (anti-RA) benefits, as is very well documented in the literature [114,115]. Phenolic acids, which are plant metabolites found in many different types of plants, also have anti-rheumatoid-arthritis benefits. Comprehensive investigations of ferulic acid have revealed significant findings about its impact on the pathogenic pathway of RA, with a specific focus on the interplay between RANKL, an osteogenic factor, and the NF-κB signaling pathway [116] (Figure 5). One group conducted a limited investigation on chlorogenic acid and found that the use of osteoclasts as the sole cell line in their experiment was restrictive due to several immunological components in the pathological pathway of RA [117]. Another group conducted a study investigating the impact of p-coumaric acid (CA) on the RANKL system and its interaction with components in the T-cell immune system, and the results were found to be promising [118].

Stilbenes are polyphenolic compounds characterized by the presence of two phenyl groups linked by a two-carbon methylene bridge. The majority of studies investigating the anti-RA benefits of stilbenes have focused on resveratrol. Three prominent works of research have been conducted on resveratrol. In one study, the authors specifically observed the involvement of resveratrol in the regulation of the interaction between COX-2 and PGE2 [114]. This study is distinctive because it specifically examines the impact of particulate matter (PM) from air pollution on RA, and investigates the influence of resveratrol on the inflammatory pathways associated with PM-induced RA [119]. Another study group examined the impact of this chemical from three different viewpoints. These researchers noted alterations in the immune system, systemic inflammation, and oxidative stress [120]. The third study investigated the effectiveness of resveratrol in inhibiting neutrophil extracellular traps (NETs) that cause joint hyperalgesia in C57BL/6 mice after adjuvant-induced arthritis (AIA). The mice had higher levels of NETs in their joints and increased expression of the PADI4 gene. Treatment with resveratrol significantly inhibited joint hyperalgesia, increased the mechanical threshold, decreased edema, decreased inflammatory cytokine production, increased COX-2 expression, and decreased NF-κB immunostaining (Figure 5). This study suggested that resveratrol reduces inflammation mediated by PADI4 and COX-2, potentially treating joint pain in RA [121].

It has been shown that the *Zingiber roseum* (*Z. roseum*) leaf methanol extract (ZrlME) demonstrates efficacy in treating various ailments. The extract demonstrated strong dose-dependent analgesic efficacy, and induced higher anti-inflammatory activity and a significant reduction in rectal temperature. Seven polyphenolic metabolites were identified, with strong binding affinities and significant COX-2 inhibitory activity. The polyphenols were found to be nontoxic and exhibited antioxidant, analgesic, anti-inflammatory, antipyretic, and hepatoprotective properties. This research confirms the traditional use of *Z. roseum* for various ailments [122].

OA is a prevalent and persistent degenerative disease characterized by chronic inflammation that impacts a large number of individuals globally [123]. This condition is marked by gradual and ongoing deterioration and loss of the cartilage in the joints, as well as the surrounding muscles. It is also accompanied by the growth of bony outgrowths, inflammation of the synovial membrane, and the degeneration of ligaments, the subchondral bone, menisci, and the infrapatellar fat pad. These factors contribute to the development of osteophytes, subchondral sclerosis, bone cysts, and a reduction in the space within the joint [124].

In a rat model of OA, some researchers demonstrated that quercetin might diminish the levels of pro-inflammatory cytokines (IL-1β, IL-18, and TNF-α) via inhibiting the IRAK1/NLRP3 signaling pathway. The in vivo validation of this occurred when quercetin reduced oxidative stress, inflammation, apoptosis, and cartilage degradation in rat chondrocytes induced by IL-1β by suppressing the expression of IRAK1, NLRP3, iNOS, COX-2, and caspase-3 [125]. Pursuant to these outcomes, the authors further illustrated that quercetin displays chondroprotective effects by diminishing chondrocyte apoptosis and cartilage degradation in the rat OA model [126]. This study found that IL-1β-induced rat chondrocytes had their matrix degradation reversed, their expression of the proteases involved in matrix degradation downregulated, and their caspase-3 pathway expression suppressed. Furthermore, their synovial fluid showed increased levels of transforming growth factor-β (TGF-β2). In addition to enhancing glycosaminoglycan synthesis and creating a pro-chondrogenic milieu for chondrocytes, quercetin stimulated the M2 polarization of macrophages, which in turn improved cartilage regeneration [123,126]. At a concentration of 100 µmol/l, quercetin was noted to obstruct the p38 MAPK signaling cascade and ADAMTS, thereby reducing the inflammatory factors associated with OA. Furthermore, a recent study conducted by Wang and collaborators [127] revealed that quercetin enhanced the production of COL-II, thereby supporting cartilage repair mechanisms [127]. In a mouse model of OA caused by monosodium iodoacetate (MIA), quercetin showed its anti-OA effects via inhibiting matrix metalloproteinases (MMPs), as shown by a marked decrease in the blood concentrations of MMP-3 and MMP-13 [128].

The stilbene resveratrol (Figure 3) is another type of polyphenol that is prevalent in peanuts, grape skins, and Japanese knotweed (*Reynoutria japonica*) [129]. Resveratrol has been the subject of comprehensive examination within the nutraceuticals domain due to its potential in the management of degenerative conditions like OA. This efficacy is associated with its ability to regulate crucial pathways related to the signaling of oxidative stress, as evidenced in multiple research studies [130,131,132,133]. When it comes to controlling symptoms and pain associated with knee OA, two recent clinical investigations have shown that resveratrol plays a role [132]. Consuming a daily dosage of 500 mg of resveratrol resulted in a notable reduction in pain and an elevation in aggrecan serum levels. Nonetheless, there was no considerable drop seen in the blood levels of IL-6, IL-1β, and TNF-α [134]. Resveratrol and its biochemical precursor, polydatin, can diminish the development of ROS, nitrogen oxides (NOx), and the courier RNAs for interleukin-1 (IL-1) and interleukin-1 beta (IL-1ß) provoked by monosodium urate and calcium pyrophosphate crystals, as per an in vivo investigation on the THP-1 monocytic cell line. Calcium pyrophosphate crystals, which can be seen in OA joints and appear to have a role in synovial inflammation, can promote inflammation, and this investigation also showed that resveratrol and its precursor can diminish this inflammation [130]. While a curcuminoid-rich extract can alleviate discomfort associated with OA, it has no effect on cartilage composition or effusion–synovitis in the knee, according to clinical trials [127]. A separate trial investigated the analgesic efficacy of curcuminoids plus diclofenac, finding that pain was minimized and side effects were greatly diminished as the functional capacity increased [135]. One group of scientists created and evaluated a surface-managed, water-scattering turmeric formulation (Theracurmin) for individuals with OA to improve the availability and effectiveness of turmeric [136]. Over three-quarters of these OA patients reported a decrease in pain while taking 180 milligrams of curcumin (Figure 3) or Theracurmin daily [136]. Curcumin has been demonstrated to mitigate OA and safeguard bone wellness. Its chondroprotective properties diminish apoptosis in chondrocytes following IL-1β stimulation, restraining the p65 promoter activity of NF-κB. A nanostructured form of curcumin was found to exhibit defensive impacts on articular cartilage in an OA model, suppressing pro-inflammatory cytokines [137]. Although oral curcumin decelerates OA advancement, it does not notably diminish pain. Curcumin nanomicelles improved OA symptoms in knee patients over six weeks. Curcumin, prepped onto adipose-derived mesenchymal-stem-cell-derived small extracellular vesicles, exhibited improved effectiveness and cartilage-protective impacts against osteoarthritis [137]. There have been limited investigations conducted in the previous five years to ascertain if green-tea polyphenols, like EGCG (Figure 3), may retard cartilage degeneration and alleviate joint discomfort linked with OA. As previously observed in vivo, oral treatment with EGCG in mice significantly reduced inflammatory symptoms of gout in an acute gout model [138]. Another team of researchers discovered that, in comparison to a control cohort that only received diclofenac, the experimental cohort that received green-tea polyphenols alongside diclofenac encountered notably reduced unease. Nevertheless, there was no substantial variance between the treated and control cohorts in terms of knee rigidity [139]. The previously mentioned investigations have assessed numerous notable polyphenols and polyphenol-rich extracts in various models of rheumatoid and osteoarthritis. The majority of these studies suggest that polyphenols offer analgesic effects and enhance operational capability in models of arthritis. Their main aim was to scrutinize the mechanisms that contribute to the favorable influence of these treatments on the pathophysiological processes involved in arthritis progression. Undoubtedly, conducting additional, well-structured clinical trials could establish a foundation for targeted therapies using polyphenols in individuals with arthritis.

### 2.4. Anti-Microbial Activity

**Figure 6:**
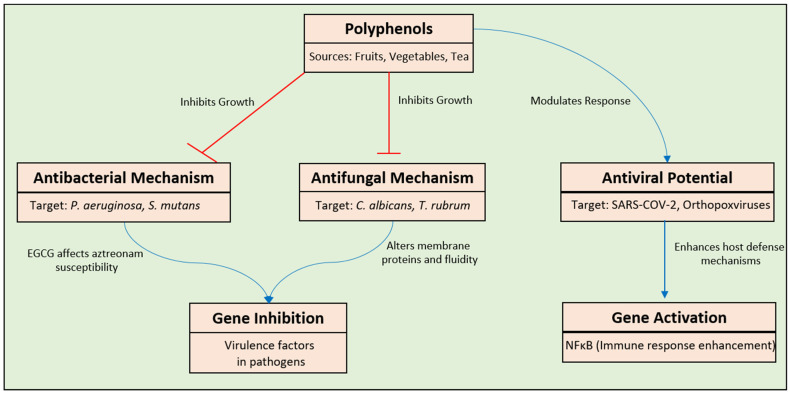
The protective effects and action mechanisms of dietary polyphenols against microbial infections.

Polyphenols have been acknowledged as powerful inhibitors of the reproduction of various bacterial and fungal pathogens, encompassing both Gram-positive and Gram-negative bacteria [140]. The urgent need for effective antibiotics targeting *Pseudomonas aeruginosa* (*P. aeruginosa*) is an additional concern within healthcare environments. A multitude of research studies have illustrated the effectiveness of polyphenols and polyphenolic extracts in combating this bacterium. One of the pharmacological agents that shows activity against clinical strains of *P. aeruginosa* is epigallocatechin gallate (EGCG) [141]. The reinstatement of susceptibility to aztreonam by EGCG reached a level equivalent to or below the threshold established by the European Committee for Anti-Microbial Susceptibility Testing (Figure 6). Utilizing combination therapy in *Galleria mellonella* (*G. mellonella*) displayed superiority over monotherapy, leading to enhanced larval survival rates (94% compared to a maximum of 63%) [141].

Dental caries (DC) represents a prevalent oral pathology instigated by *Streptocoque mutans* (*S. mutans*) bacteria. In an effort to mitigate the risk of DC, compounds inhibiting the growth and virulence factors of *S. mutans* were investigated. An examination was completed on three tannins (often recognized as tannic acid) demonstrating powerful antibacterial effects. The findings indicated significant antibacterial efficacy against *S. mutans*, attributed to alterations in membrane fluidity and interactions with membrane proteins (Figure 6). These compounds hold promise as potential natural preventive measures for DC [142]. Another research endeavor scrutinized the antioxidant and antibacterial attributes of phenolic extracts derived from solid residues (SRs) originating from the essential oil industry. The fruit extracts obtained from Greek oregano (*Origanum vulgare*), rosemary, spearmint (*Rosmarinus officinalis* L.), lemon balm (*Melissa officinalis*), and Greek sage (*Salvia fruticosa*) were evaluated for their extensive phenolic contents, antioxidant capacities, and antibacterial effects on various Gram-positive strains. The findings propose the plausible utilization of extracts from post-distillation remnants of medicinal and aromatic plants as anti-microbial agents within the food industry [143]. These extracts could potentially be incorporated into bread dough to impede the proliferation of *Bacillus* strains responsible for ropiness [143]. One research group investigated the chemical composition and biological activities of Moroccan *Lactuca saligna* extracts. These scientists characterized the polyphenolic compounds present in the hydro-methanolic extracts and assessed their antioxidant and antibacterial effects [144]. A total of 29 phenolic compounds were identified, including dicaffeoyltartaric acid, luteolin 7-glucuronide, 3,5-di-O-caffeoylquinic acid, and 5-caffeoylquinic acid. The extracts also showed remarkable antibacterial activity against *Escherichia coli* (*E. coli*), *Salmonella typhimurium* (*S. typhimurium*), *P. aeruginosa*, *Enterococcus faecalis* (*E. faecalis*), *Staphylococcus aureus* (*S. aureus*), and *Listeria monocytogenes* (*L. monocytogenes*). Computational analyses pointed out that these compounds could function as optimal candidates for the production of groundbreaking antibacterial agents. Further investigations through in vitro and in vivo studies are essential to elucidate the primary biological effects of these plants [144,145]. A literature review compiling diverse polyphenols effective against *S. aureus* strains indicated that flavonoids were particularly potent against clinical isolates. These flavonoids included flavonols like morin and kaempferol, as well as flavanols and derivatives like epigallocatechin gallate, catechin acyl derivates, epicatechin gallate, 3-O-decyl-catechin, catechin, and phenolic acids and their derivatives like protocatechuic acid ethyl ester and caffeic acid [140,146]. When identifying antibacterial polyphenolic medications with minimal cytotoxicity and limited adverse effects, these compounds could represent an optimal choice. Research on the antibacterial properties of polyphenols and extracts high in polyphenols against various bacteria, including both Gram-positive and Gram-negative strains, has taken big hit recently. Research conducted previously has highlighted that polyphenols and extracts have the ability to restrict the proliferation of specific yeast strains, thus indicating potential antifungal properties. Antifungal effects against *Candida albicans* (*C. albicans*) were demonstrated with extracts of *Acacia nilotica* (*A. nilotica*), *Cinnamomum zeylanicum* (*C. zeylanicum*), and *Syzygium aromaticum* (*S. aromaticum*) [147]. Furthermore, Marinaş and collaborators [148] found that extracts derived from the vegetative parts and leaves of different *Amaranthus retroflexus* (*A. retroflexus*) species exhibited efficacy against *Candida famata* (*C. famata*), *Candida utilis* (*C. utilis*), *C. albicans*, and *Saccharomyces cerevisiae*, particularly displaying a pronounced impact on *C. famata*. Moreover, these extracts underwent testing in combination with antibiotics to evaluate their synergistic effects, yielding positive outcomes. *Candida species* and *Candida neoformans* (*C. neoformans*) were effectively inhibited by gallic acid (Figure 3), ellagic acid, and corilagin. Extracts of longan (*Dimocarpus longan* L.) and ellagic acid from seeds inhibited the growth of *C. albicans* and *C. neoformans*. The longan seed extract showed a slight inhibitory effect on the analyzed species of dermatophytes, although corilagin and ellagic acid displayed restricted antifungal activity against *Trichphyton rubrum* (*T. rubrum*), *Microsporum gypseum* (*M. gypseum*), and *Epidermophyton floccosum* (*E. floccosum*) [149]. The results suggested that the utilization of this longan seed extract and its polyphenolic components as an antifungal agent in oral care products could be effective in treating opportunistic yeast infections. It has also been noted that the simultaneous employment of azoles with trans-resveratrol (t-RSV) yielded a synergistic outcome on certain *C. albicans* strains under inspection. Moreover, when administered separately, t-RSV did not exhibit any antifungal efficacy [140,150]. Soil samples were searched for myxobacteria, using 30 clinical isolates of *C. albicans*, and the scientists calculated the MIC50 values of fluconazole, itraconazole, and ketoconazole. When tested with t-RSV, the combination of the two drugs showed synergy against over 83% of the clinical strains. When experimenting with various cultural media, the authors discovered that the synergy changed. Even azole-resistant isolates were able to be effectively treated with t-RSV. Among the three fluconazole-resistant strains of *C. albicans* that were examined, t-RSV exhibited the power to notably elevate the antifungal susceptibility of two of these strains. This suggests that when t-RSV alone is ineffective, using it in combination with other azole medications is an alternative method [150]. The strain exhibiting resistance was subsequently subjected to treatment using itraconazole and ketoconazole alongside t-RSV, leading to the observation of antifungal outcomes.

Monkeypox outbreaks present a worldwide health risk, which is made worse by the lack of effective medications for orthopoxviruses. Molecular modeling, with a specific focus on natural ingredients such as traditional Chinese medicine (TCM), has identified potential inhibitors. Four substances, namely rosmarinic acid, myricitrin, quercitrin, and ofloxacin, have exhibited a substantial affinity for monkeypox DNA topoisomerase I, indicating potential antiviral properties. The stability of this system has been confirmed through simulations of molecular dynamics [151,152]. Research conducted by another group has underscored the potential use of these chemicals as inhibitors of poxviruses, emphasizing the necessity for additional investigations to evaluate their therapeutic effectiveness [153]. One very important study focused on isolating and identifying chemical compounds derived from *Agaricus blazei* (*A. blazei*) Murrill and studying their antifungal properties in a laboratory setting [154]. This study aimed to explore the antifungal properties of *A. blazei*, a fungus renowned for its therapeutic applications and dietary consumption. Six compounds were isolated from *A. blazei*: linoleic acid, 1,1′-oxybis(2,4-di-tert-butylbenzene), glycerol monolinoleate, volemolide (17R)-17-methylincisterol, (24s)-ergosta-7-en-3-ol, and dibutyl phthalate [154]. These compounds were assessed against various fungal strains: *Trichophyton mentagrophytes* (*T. mentagrophology*), *T. rubrum*, *C. albicans*, and *C. neoformans*. Compound 2 exhibited substantial suppression against *T. mentagrophology*, with compound 3 exhibiting substantial suppression against *T. rubrum* and compound 6 exhibiting substantial suppression against *C. albicans*. The results of this study emphasize the therapeutic capabilities of *A. blazei* as an antifungal substance, indicating intriguing directions for future investigations [152,154]. A comprehensive analysis of the existing literature shows that research significantly emphasizes the therapeutic benefits of polyphenol extracts obtained from *Geraniaceae sanguineum* (*G. sanguineum*) [155]. The polyphenols identified in *G. sanguineum* showcase notable anti-inflammatory, antioxidant, and antiviral attributes, positioning it as a promising candidate in the arena of natural therapeutic interventions. *G. sanguineum* possesses a distinctive characteristic whereby it can impede virus replication by inhibiting the activity of DNA polymerase and reverse transcriptase enzymes [156]. The direct antiviral impact of this substance works together with its immunomodulatory qualities, which helps to resolve infections. The inclusion of condensed tannins within *G. sanguineum* is pivotal in mitigating lung damage associated with respiratory viral infections, potentially including its effects on COVID-19. These tannins exhibit effectiveness by suppressing the activity of proteases in the lungs and regulating the reactions of macrophages, thereby providing a versatile defense against respiratory viruses [155,156]. Ellagitannins and ellagic acid are two of the several polyphenolic metabolites present in pomegranate plants (*Punica granatum* L.). Traditional medicine makes use of this plant, and research has shown that its isolated compounds can aid the body’s immune response to viral infections and subsequent recoveries by reducing inflammation and boosting antioxidant levels [140]. Recent investigations propose that the favorable impacts of pomegranate polyphenol extracts and their ellagitannin constituents and byproducts pertain to the control of the NF-κB pathway, the handling of immune cell infiltration, and the regulation of cytokine secretion, as well as of reactive oxygen and nitrogen species. Various viruses, such as SARS-CoV-2, are curbed in their capability to infect by the interactions between pomegranate extracts and ellagitannins in vivo. Ellagitannins have the ability to form complexes with numerous human and SARS-CoV-2 proteins, particularly proteases, as evidenced in computational docking studies [157]. More research, both in vitro and in vivo, is required to better understand the interactions among polyphenols, viruses, and their hosts. To effectively address the SARS-CoV-2 virus, it is essential to restrict the inflammatory reaction of the host towards viral infections and replenish reduced antioxidant levels post COVID-19 recovery (Figure 6). Research indicates that extracts from pomegranates, ellagitannins, and ellagic acid have shown promise as therapeutic agents in this particular context [140,158]. Despite encouraging results from several investigations, as highlighted above, additional research is required to establish the efficacy of polyphenols in managing and avoiding microbial disorders affecting humans. A proactive strategy in environmental conservation to curb the proliferation of such illnesses may involve the utilization of polyphenol-based anti-microbial therapies. A potential remedy to combat antibiotic resistance is the development of topical pharmaceutical compositions incorporating polyphenols to enhance the efficiency of current antibiotics utilized in medical practice. Given the ongoing emergence of resistance patterns and the ambiguous outlook on existing therapies, it is prudent to formulate the aforementioned compositions and execute clinical trials to assess the viability of these proposed antibiotic alternatives. These procedures are imperative prerequisites prior to the integration of the suggested alternatives into mainstream medical applications.

### 2.5. Cardioprotective Activity

Cardiovascular diseases (CVDs) are perceived as a primary contributor to worldwide fatalities, involving concerns such as hypertension, arterial hardening, heart attacks, irregular heart rhythms, and heart failure [159,160]. Emerging research has illuminated a noticeable surge in the prevalence of heart ailments [159,160,161]. It is essential to understand that the medications often employed for cardiovascular diseases (CVDs), including statins, angiotensin-converting enzyme inhibitors (ACEIs), angiotensin receptor blockers (ARBs), fibrates, and β-blockers, pose a risk of causing unwanted side effects. Therefore, there exists a pressing necessity to investigate and formulate novel therapies for cardiovascular diseases [162]. As per the existing research, maintaining a healthy, well-balanced diet can greatly decrease the chances of developing cardiovascular diseases [163]. Diets that are vegetarian or predominantly plant-based have been shown to not only lead to an increased life expectancy but also decrease the prevalence of cardiovascular conditions [164]. In addition, data from epidemiological studies propose that following a diet abundant in polyphenols could potentially lower by fifty percent the probability of developing cardiovascular diseases [165]. An investigation conducted recently delved into the comprehension and consciousness of Romanian individuals in relation to cardiometabolic hazards (CMHs) and the advantageous impacts of polyphenols. The outcomes unveiled that around 80% of the respondents exhibited apprehension regarding their well-being and dietary selections, although this sentiment varied depending on factors such as age, level of education, and body mass index. Despite this high level of health concern, only 35% demonstrated a substantial understanding of polyphenols. While 86% were aware of their antioxidant properties, only 26% were familiar with their prebiotic effects. These results highlight the necessity for specific educational initiatives aimed at enhancing people’s knowledge and individual practices concerning CMH factors and the advantages of polyphenols [166]. Atherosclerosis represents a pathological condition characterized by the progressive calcification and constriction of blood vessels as a result of the gradual accumulation of lipids, cholesterol, and various other substances on and within the arterial walls. The initiation of this process occurs when lipids breach the endothelial layer and undergo oxidation facilitated by endothelial smooth muscle cells and activated macrophages [167,168]. An important element contributing to the escalation of cardiovascular diseases (CVDs) is atherosclerosis, which emerges from dyslipidemia and an inflammatory state.

**Figure 7:**
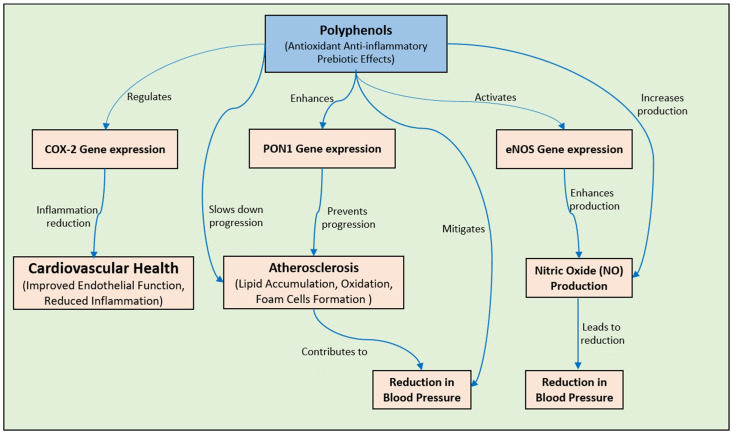
The protective effects and action mechanisms of dietary polyphenols regarding cardiovascular health.

Paraoxonase enzymes, notably paraoxonase 1 (PON1), exert a pivotal role in safeguarding against diverse ailments, including CVDs (Figure 7). The manipulation of PON1 expression could be a viable therapeutic target, with lifestyle modifications like dietary adjustments and physical activity potentially augmenting its levels. Nevertheless, additional investigations are imperative to scrutinize the effects of herbal constituents and flavonoids on the functionality of PON1 [169]. The formation of ROS and reactive nitrogen species (RNS) can accelerate the oxidation of LDL cholesterol. This process is exacerbated by the accumulation of macrophages in the affected area, which facilitates the clearance of oxidized LDL cells and their transformation into foam cells. Atherosclerotic plaques exhibit a backdrop of inflammation marked by endothelial dysfunction, foam cell accumulation, vascular smooth muscle proliferation, and an elevated population of monocytes and macrophages within the vascular intima. The upsurge is instigated by adhesion molecules and chemokines [170]. This increase is triggered by adhesion molecules and chemokines. Moreover, the aggregation of the extracellular matrix encircling the inflamed area results in plaque development, which hinders blood vessel passages. Consequently, this leads to a compromised natural relaxation ability of the blood vessels, culminating in functional impairment [162,169,170]. The plausible effects of polyphenols on atherosclerosis have been the area of several research inquiries. Several studies have proven that red and purple grape juice could potentially delay the initiation and progression of atherosclerosis. The role of NO, produced by endothelial cells, in regulating blood pressure and vascular tone is extensively acknowledged. NO activates a series of events in the smooth muscle cells of arteries, known as the cGMP–protein kinase G pathway [162]. This process of activation initiates the opening of potassium channels, resulting in membrane hyperpolarization and the suppression of intracellular calcium influx, the latter leading to vasodilation. In contrast, protein kinase G phosphorylates myosin light chains to reduce vasoconstriction in artery smooth muscles [171]. The interaction between polyphenols and the endothelium, primarily through NO production, is considered a significant discovery [172,173]. Research indicates that hypertensive individuals can lower their blood pressure by using olive oil [174]. Furthermore, polyphenols like resveratrol, EGCG, and quercetin enhance endothelium-dependent relaxation by regulating the gene expression of inducible NO synthase and COX-2 (Figure 7), thereby reducing the risk of cardiovascular diseases [175]. Multiple elements, such as trans-resveratrol, a polyphenol, raise NO production via a calcium ion-dependent pathway in endothelial cells, improving vasorelaxant effects and potentially enhancing cardiovascular health [176]. By blocking Ca2+ ATP-ase in endothelial cells or opening potassium channels, resveratrol and quercetin raise the intracellular ion concentration of calcium Ca^2+^ ions [177].

The polyphenol-rich blackcurrant extract (BCE) significantly upregulates eNOS mRNA levels (Figure 7) and NO synthesis through phytoestrogenic activity, thereby promoting blood vessel health in OVX rats as a postmenopausal model [178]. Chocolate procyanidins have displayed the capacity to diminish the leukotriene–prostacyclin ratio in both individuals and human aortic endothelial cells, suggesting a potential impact on the cardiovascular health benefits they offer. Research suggests that flavanols and procyanidins derived from cocoa block the generation of 15-hydroxy-eicosatetraenoic acid, affecting the lipoxygenase pathway [179]. Additionally, within scientific studies, cocoa procyanidins, notably the pentameric and octameric fractions, have been observed to impede the expression of the tyrosine kinase ErbB2. This identified inhibition is integral to modulating both endothelial cell proliferation and angiogenic signaling pathways [180,181]. Furthermore, specific cocoa procyanidins, such as procyanidin B2, have been identified as inhibitors of thrombin-induced activation and the expression of matrix metalloproteinase-2 in vascular smooth muscle cells. This discovery suggests potential antiatherosclerotic effects [182]. Collectively, these findings suggest that chocolate procyanidins play a role in modulating key pathways related to cardiovascular health and vascular function. The inflammatory response to injury is a complex biological process that reacts to harmful stimuli. Several enzymes, like cyclooxygenase (COX), lipoxygenase (LOX), tyrosine kinase (TK), phospholipase A2 (PLA2s), and protein kinase C, play an important role in the regulation of the inflammatory response [159,160,161,162]. Some specific flavonoids have been shown to directly inhibit these enzymes, thereby directly impacting inflammation [183,184]. Nutrition plays a significant role in the prevention and management of chronic inflammation, as emphasized in epidemiological studies. Through ex vivo and in vivo models, scientists have identified certain flavonoids that demonstrate anti-inflammatory characteristics. These flavonoids significantly influence the creation of prostaglandins, a crucial biological function. Numerous in vivo investigations have demonstrated that hesperidin and diosmin, which are flavonoids present in citrus fruits, possess the capability to reduce the synthesis of prostaglandins [185]. The production of leukocytes is recognized as a crucial phase in the advancement of inflammation observed in cardiovascular diseases and other ailments. Arachidonic acid synthesis ultimately leads to the production of cytokines (IL-1) and chemokines (IL-8) by neutrophils [161,162]. This process is facilitated by both COX and LOX enzymes. Quercetin is particularly efficient at inhibiting the production of prostaglandins (PGs), leukotrienes (LTs), and thromboxanes (TXAs) by blocking the enzymes COX and LOX [186,187,188]. Multiple ex vivo experiments have provided evidence that certain flavonoids, such as bilobetine, morelloflavone, amentoflavone, and those present in Sophora flavescens, exert their impact by inhibiting the synthesis of arachidonic acid [189]. Moreover, resveratrol is considered a compound with anti-inflammatory properties, as it blocks the synthesis of PGs [190].

It may be concluded that polyphenols have a wide range of effects on the intricate pathophysiology of CVDs. These influences encompass reductions in blood pressure and cholesterol levels, the mitigation of inflammation, and the facilitation of endothelial function recovery. However, polyphenols cannot be used in therapeutic settings due to several major obstacles. These include potential short- or long-term negative effects on human beings, as well as concerns regarding the treatment’s dosage, specificity, potency, and feasibility. The distribution and target cells of natural polyphenols determine their safety and whether they have any negative effects on the body, although they are typically considered safe. To overcome these obstacles, more human intervention trials, large-scale cohort studies, and animal research trials are needed in the future. 

### 2.6. Neuroprotective Activity

**Figure 8:**
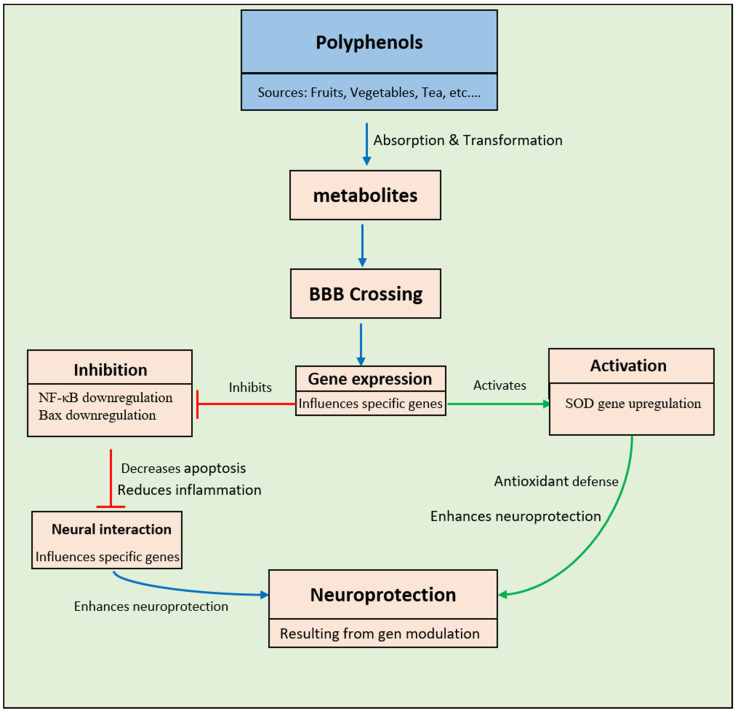
The protective effects and action mechanisms of dietary polyphenols against neurodegenerative disorders.

The worldwide population of elderly individuals is forecasted to multiply within the following thirty years, resulting in a rise in the frequency of neurological ailments [191,192,193]. Neurodegenerative illnesses constitute persistent ailments that affect the central nervous system. Examples include Alzheimer’s disease (AD), Parkinson’s disease (PD), dementia with Lewy bodies (DLB), multiple system atrophy (MSA), progressive supranuclear palsy (PSP), and Huntington’s disease (HD) [193,194]. Aging is a fundamental element in the process of neurodegeneration, resulting in alterations in the brain’s tissue equilibrium and playing a role in the initiation of neurodegenerative conditions. Tailored attention is crucial for individuals with neurodegenerative diseases as conventional therapies are ineffective for the vast majority of individuals. The absence of achievement in pharmacological investigations emphasizes the requirement for innovative treatment strategies, one of which might entail the utilization of polyphenolic substances [193,194]. Research indicates that phenolic compounds, when ingested, have the intrinsic capacity to inhibit enzymes involved in glucose metabolism, notably α-glucosidase and α-amylase [193,195]. Moreover, a variety of phenolic compounds have the ability to penetrate the blood–brain barrier (BBB) (Figure 8) and display noteworthy physiological impacts in experimental setups representing neurodegenerative disorders, both in laboratory settings and within living organisms. 

The impacts observed are mainly ascribed to the antioxidative and anti-inflammatory characteristics of polyphenols, as reported by Lopes and collaborators [196]. In cases where there are changes to the BBB, they can even enhance its permeability [192]. Since polyphenols can diffuse across the BBB in the form of aglycones or their conjugation products, even at low concentrations in the brain (1 nmol/g of tissue), they are thought to impact neuronal circuits [197]. The capacity of flavonoids to navigate the BBB is determined by two important elements: their lipid-soluble nature and their conjugation ability. Metabolites undergoing methylation in the small intestine and liver exhibit a decrease in polarity and an elevation in lipophilicity. This transformation aids in enhancing their permeability across the BBB compared to their original aglycones, as indicated by Arias-Sánchez and collaborators [197]. To traverse the BBB, polyphenols with lower lipophilicity are required to adhere to particular ATP-dependent transporters. In the intricate network of signal transduction pathways involving multiple kinases, including MAPK, PI3K, and PKB, polyphenols exhibit the capacity to interact directly with neurotransmitter pathways [197,198,199,200].

The potential of polyphenols to prevent and help treat neurodegenerative diseases caused by aging has been the subject of intensive research over the last several decades. Therefore, certain polyphenols exhibit pleiotropic impacts on neuronal cells and have been documented to influence neuronal functionality [201,202,203,204]. Studies have demonstrated that consuming foods high in polyphenols can help preserve cognitive skills by promoting neuronal survival, differentiation, and regeneration [37,197,205]. In addition, polyphenols can significantly slow the progression of neurodegenerative diseases by improving learning, memory, and cognition [206]. In AD, flavonoids have a neuroprotective effect that is connected to the mediation of GSK3β and CDK5 [207]. The second benefit comes from a mechanism that directly protects neurons. However, polyphenols also provide indirect protection to neurons through their modulation of the gut microbiota’s composition and the metabolites discharged into the bloodstream, along with other pathways. These two mechanisms lead to modifications in neurotransmitter and neuropeptide production, ultimately affecting brain functions [197,199,208]. The application of phenolic compounds, such as mangiferin and morin, has been recognized as a means to facilitate neuroprotection in experimental models of neuronal injury caused by excitotoxicity, stemming from the excessive activation of N-methyl-D-aspartate (NMDA) receptors. Neuroprotection is attained through the activation of the antioxidant enzyme system, the inhibition of ROS generation, and the reinstatement of mitochondrial membrane potential, as indicated in [193]. These compounds are also linked to the regulation of cytosolic levels of Bax and the translocation of NF-κB (Figure 8), leading to decreased neuronal death and inflammation. Additionally, they influence the protein kinase B (AKT) and extracellular signal-regulated kinase (ERK) 1/2 pathways, which are essential for neuronal survival, as validated by Campos-Esparza and colleagues [209]. The findings suggest a correlation between phenolic compounds and the modulation of neuronal apoptosis through the stimulation of important proteins such as Bax, NF-κB, AKT, and ERK 1/2, which play critical roles in processes related to inflammation and cellular survival. Further benefits of polyphenols include a decrease in oxidative stress and neuroinflammation, as well as alterations to cell death mechanisms brought about by low-molecular-weight phenolic compounds such as catechol-O-sulfate and pyrogallol-O-sulfate, which are present in plasma after consuming polyphenol-rich foods [193]. The following genes were upregulated in a 3D model of human PD cells treated with polyphenol: FTH1, AKT1, BCL2L1, AUTOPHY (ATG5, ATG12, BECN1), and UPR (ATF4, ATF6, DDIT3, CALR, HSPA4, HERPUD1). Furthermore, polyphenols regulate pathways associated with cellular senescence, including IL-7, JAK/STAT, PI3K/AKT, and PPAR-α [210]. The change of microglia to an inflammatory phenotype is an essential part of neurodegeneration. Apigenin reduces histological inflammatory markers (Iba-1+) and the number of Iba-1+ cells following chronic therapy in male and female Wistar rats, according to a chronic neuro-inflammatory paradigm involving glial fibrillary acidic protein–interleukin 6 (GFAP-IL6) [211]. Aligned with earlier research discoveries, this investigation validates the impact of polyphenolic compounds on modulating the maturation of microglial or immune system cells, alongside diminishing inflammation and ROS. Enzymes are responsible for controlling ROS within cells. Superoxide dismutase (SOD) is not categorized under the metalloenzyme group, which is not indispensable for the antioxidant defense systems, as described by Saxena and collaborators [212]. The potential of hydroxyl groups found in polyphenolic compounds to offer protons and electrons leads to two primary outcomes: initially, they impede or delay the oxidation of organic substances, and subsequently, they alleviate the oxidation caused by ROS [193,213]. Given that oxidative stress is a major cause of cellular damage, these compounds could be promising candidates for alternative therapies. One group of scientists identified that after six months of undergoing curcumin therapy, subjects suffering from amyotrophic lateral sclerosis showed reduced levels of specific indicators of oxidative stress, such as ferric-reducing ability, lactates, total thiols, and oxidative protein products [214]. The carrying of flavonoids by serum albumin following the consumption of flavonoid-packed food impacts their bioavailability, as indicated by Dufour and Dangles [215]. Another benefit of this interaction is the reduced breakdown of these molecules. The antioxidant potential of quercetin may be enhanced by the fact that it degrades more rapidly when bound to albumin than when isolated in oxygen-dependent activities [216]. These findings suggest that phenolic compounds may protect cells from ROS-induced damage. The necessity of investigating polyphenolic compounds as a medication or adjuvant to control neurodegenerative illnesses is underscored by the limited number of clinical trials involving patients with these conditions [193,197]. Some researchers investigated the correlations between total polyphenol intakes and neuropsychological test scores measuring cognitive function among 2574 middle-aged adults. This research showed that consuming polyphenols, in general, improves memory performance, and that consuming specific types, such as catechins, theaflavins, flavonols, and hydroxybenzoic acids, in particular, has a favorable effect on memory performance [217]. By widening the window of opportunity for recombinant tissue plasminogen activator (rt-PA) therapy, an adjuvant used to treat ischemia, fisetin helped patients suffering from ischemic stroke [193]. The expression of cognitive dysfunction was milder in subjects treated exclusively with fisetin in combination with a plasminogen activator treatment. This was associated with lower levels of essential proteins for tissue restoration, particularly matrix metalloproteinase (MMP) 2, matrix metalloproteinase (MMP) 9, and C-reactive protein (CRP) [218]. In an intriguing study conducted by Moussa and collaborators [219], a group of 119 patients diagnosed with mild to moderate Alzheimer’s disease underwent a year-long treatment regimen involving resveratrol, a compound renowned for its potential health benefits. Administered orally at doses of up to 1 g twice daily, this treatment was studied to investigate resveratrol’s impact on Alzheimer’s pathology. One of the key assessments conducted focused on the quantification of Aβ40 within the cerebrospinal fluid, a pivotal indicator in the advancement of Alzheimer’s disease, representing the final metabolite of the amyloid precursor protein. Notably, the research findings indicated a noteworthy decrease in Aβ40 concentrations, implying a favorable therapeutic outcome.

Furthermore, the examination demonstrated the effectiveness of resveratrol in controlling inflammation in the brain, a significant aspect in the progression of neurodegenerative conditions. This was evidenced by the decreased levels of inflammatory markers such as IL-12P40, IL-12P70, and C-C motif chemokine ligand 5 (CCL5). The results revealed in this research provide compelling evidence that resveratrol possesses the ability to cross the blood–brain barrier, establishing a new pathway for treating inflammation in Alzheimer’s disease and potentially other degenerative brain conditions. 

The beneficial effects of a diet rich in polyphenols on symptoms associated with neuropsychiatric diseases (such as sleep dysregulation, signs of depression, and cognitive dysfunctions) are well documented [220]. It is known that the consumption of polyphenols not only reduces the severity of neuropsychiatric disorders but also acts as a mood enhancer in some cases [221]. However, results from randomized controlled trials have been inconclusive regarding the use of polyphenols in treating mental and neurological diseases, in contrast to observational human data and preclinical animal studies. Factors such as the use of isolated polyphenol compounds rather than whole foods, the duration and dosage of interventions, and the role of habitual polyphenol consumption in the diet could contribute to these discrepancies. In fact, the extent of processing applied to the polyphenols may also significantly impact their efficacy [197].

Polyphenols are regarded as beneficial substances for the prevention of neurodegenerative illnesses and present an intriguing therapeutic prospect. Studies conducted using in vitro and in vivo models indicate that polyphenols may have the potential to eliminate or reverse significant stages of AD and PD development, and even mitigate neuroinflammation. The therapeutic application of specialized metabolites is hindered by limitations, primarily due to their low solubility and bioavailability. Consequently, a growing curiosity has emerged regarding the utilization of nano-delivery systems to augment the stability and efficacy of these substances. Despite polyphenols such as flavonolic glycosides, resveratrol, and curcumin attracting considerable interest for their established multi-target effects, the neurobioactivity of many additional polyphenols is still not well understood. In order to optimize the use of edible plant specimens, it is crucial to adopt a holistic approach that integrates the analysis of nutraceutical components with the exploration of the physiological impacts of polyphenol-enriched fractions or individual compounds.

### 2.7. Anti-Obesity and Immunomodulatory Activity

**Figure 9:**
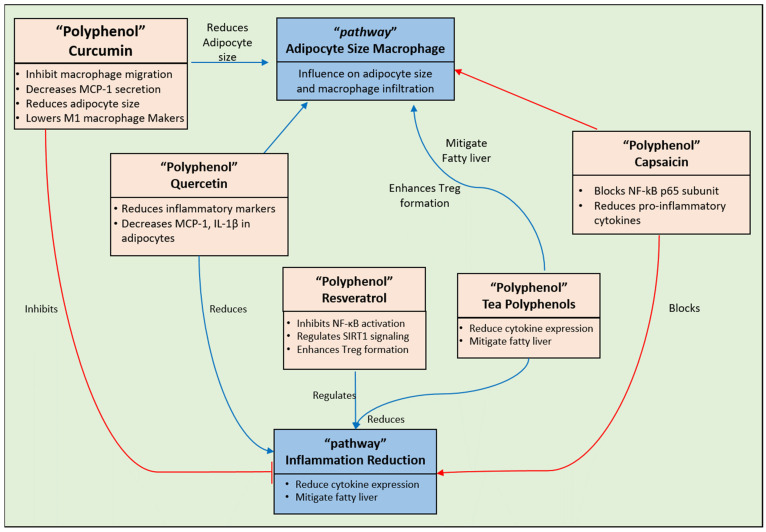
The protective effects and action mechanisms of dietary polyphenols towards anti-obesity and immunomodulatory activity.

Obesity is linked to a higher body-fat mass, which can result from various factors including genetics, environmental influences, dietary habits, lifestyle, or multiple pathophysiological clinical conditions [222,223]. It is fascinating to observe from recent research that obesity impacts both the innate and adaptive immune systems. This conclusion has been supported by various studies, highlighting a complex interaction between obesity and immune function [224,225]. The presence of several obesity-related complications, resulting from alterations in the body’s natural and acquired immune responses, leads to chronic inflammation in adipose tissue [226]. The investigation of the correlation between immune response and metabolism has gained significant traction recently. Discovering this connection could provide valuable insights into the impaired innate immunity observed in individuals with obesity. The function of polyphenols in the regulation of lipid metabolism has been the subject of thorough investigation, especially concerning obesity and cardiovascular well-being. Research has shown that the polyphenol resveratrol influences lipid metabolism by inhibiting lipogenesis and by enhancing the expression of genes related to lipid oxidation [223,227,228]. EGCG and other green-tea catechins have demonstrated lipid-lowering benefits by promoting fatty acid oxidation and reducing intestinal fat absorption [229,230]. Quercetin, a polyphenol widely present in numerous fruits and vegetables, has demonstrated the ability to impact lipid metabolism by reducing oxidative stress and inflammation, both recognized as key factors contributing to dyslipidemia and atherosclerosis [231]. In addition, curcumin, a variant of polyphenol identified in turmeric, might possess the capability to boost lipid profiles by decreasing the buildup of lipid plaque in blood vessels and stimulating the gene expression related to cholesterol metabolism [232,233]. A variety of research studies have proven the effectiveness of polyphenols in impacting lipid metabolism through mechanisms including gene expression modulation, lipid oxidation promotion, lipogenesis inhibition, and inflammation and oxidative stress reduction. Consequently, these collective effects significantly impact the management and prevention of disorders linked to dyslipidemia, including obesity and cardiovascular diseases [222,223]. Macrophages, a type of phagocyte that develops from short-lived monocytes, are responsible for removing pathogens and initiating the immune response. In the presence of an antigen, macrophages, such as dendritic cells (DCs), activate immature T-cells into effector T-cells, acting as antigen-presenting cells [234]. Macrophages are hardly ever mentioned for their crucial function in the processes of wound healing, host defense, and the regulation of inflammation. Nevertheless, their involvement may also contribute to the development of chronic diseases and conditions, such as inflammatory bowel disease, asthma, atherosclerosis, and rheumatoid arthritis. Macrophages are typically categorized into two classic phenotypes: the inflammatory M1 and the immunosuppressive M2. M1 differentiation begins with the stimulation of interferon (IFN) by bacterial lipopolysaccharides (LPSs) and the activation of Toll-like receptors (TLRs), while M2 polarization is initiated by the release of interleukin-4 (IL-4) [235]. Evidence suggests that certain cinnamon polyphenols can activate macrophages, which in turn can reduce inflammation and enhance immunological performance [236,237]. An increasingly recognized field of research, immune-cell-mediated cancer therapy focuses on leveraging cytotoxic immune cells to combat cancer. Particularly promising in this endeavor are natural killer (NK) cells, known for their ability to target both pathogens and abnormal cells within an organism. Naturally occurring cytotoxic lymphocytes, or NK cells, constitute approximately 10–15% of all blood lymphocytes. When NK cells identify cells that are “stressed”, such as those infected with a virus or a tumor, they autonomously remove those cells [238]. For a long time, scientists have explored how various plant-derived chemicals affect the ability of NK cells to combat cancer. Flavonoids, abundant phytonutrients found in many fruits and vegetables, with one subgroup, quercetin, significantly influence cytotoxic immune cells [239]. Both endogenous and exogenous chemicals that modulate the immune system can either enhance or diminish inflammation and immunological responses. The ability of natural killer cells to eliminate YAC-1 target cells is augmented by many plant secondary metabolites, including flavonoids like quercetin [240]. Reflecting the immune-modulating properties of chemicals derived from nutrition, research has shown that resveratrol affects NK cell function both directly and indirectly [241]. Resveratrol appears to enhance immune responses by altering the expression of activating cell surface receptors such as NKG2D on NK cells or by promoting the synthesis of their ligands on malignant cells [242]. Given the other chemical properties of this plant-based compound, this regulation of the immune system becomes even more intriguing. Resveratrol also suppresses the operation of usual histone deacetylases (class I, II, and IV) within human hepatoblastoma cells. Evidence points to the fact that the inhibition of HDACs can lower the growth of malignant cells in a manner that is tied to the quantity utilized [242]. Moreover, NK cell-mediated lysis was enhanced in leukemia K562 and gastric cancer SNU1 and SNU-C4 cells due to the elevated expression of various NKG2D ligands [243]. One of the major challenges in cancer treatment, yet to be overcome, is the increase in tumor resistance to radiation, chemotherapy, and targeted medications. Often, abnormalities in apoptosis are directly responsible for this increased resistance to cancer treatments; autophagy, an alternative form of cell death, may hold the key to addressing this issue [222,223,225]. Polyphenolic compounds such as rottlerin, genistein, quercetin, curcumin, and resveratrol (Figure 3) have been shown in multiple studies to mediate autophagy through various pathways. These compounds may offer new pathways for cancer treatment, which is crucial in the context of the alarming issue of drug resistance in cancer therapy [244]. Insulin resistance and other metabolic dysfunctions are exacerbated by obesity, which is defined by the complex activation of numerous inflammatory pathways. The significance of adipose tissue lies in its composition, which includes both innate and adaptive immune cells. During inflammation associated with obesity, these immune cells communicate with adipocytes (Figure 9). This interaction triggers a vicious cycle, further exacerbated by the recruitment of additional immune cells such as monocytes/macrophages, neutrophils, and T-cells, intensifying the inflammatory response [245]. Therefore, it is possible that obesity, along with its associated metabolic disorders, could potentially be managed through pharmaceutical interventions that focus on suppressing the inflammatory reactions of immune cells.

Polyphenols’ anti-inflammatory, antioxidant, and anticancer effects are well-documented pharmacological properties. Because inflammation caused by obesity may lead to an increase in pro-inflammatory mediators and immune cell buildup, polyphenolic substances may hold therapeutic promise in treating inflammatory disorders associated with obesity. Research has demonstrated that curcumin inhibits macrophage migration and polarization in the mesenteric adipose tissue and significantly decreases MCP-1 secretion from RAW264.7 macrophages fed with conditioned medium from the mesenteric adipose tissue [246] (Figure 9). Furthermore, compared to obese mice fed a high-fat diet, animals that received curcumin supplements had smaller adipocytes, showed less macrophage infiltration in their adipose tissue, and exhibited lower expressions of various pro-inflammatory M1 macrophage markers, such as CD11c, CD38, and CD80 [247]. In addition, research has shown that curcumin can inhibit NF-κB activation in adipocytes by regulating the gene expressions of C/EBPα and PPARγ outside the NF-κB pathway [248]. Resveratrol inhibits inflammatory signaling through canonical NF-κB signaling pathways, as previously demonstrated in adipocyte cell lines, aligning with prior research [249]. Resveratrol regulates SIRT1 signaling in high-fat-diet-fed mice to stabilize glucose levels and enhances Treg formation [250] (Figure 9). Moreover, capsaicin blocks the p65 subunit of NF-κB in adipocytes, mesenteric adipose tissue-conditioned media, and inhibits the MCP-1-induced migration of macrophages. Furthermore, capsaicin significantly reduces the production of pro-inflammatory cytokines by macrophages stimulated by obese-mouse mesenteric adipose tissue-conditioned medium, such as NO, TNF-α, and MCP-1 [251]. In addition, polyphenols derived from various plant extracts have shown significant promise in alleviating inflammation-related obesity. Obese mice on a high-fat diet experienced dramatic reductions in their body weight and the inflammatory gene expression in their visceral fat after consuming polyphenol-rich fractions from table grapes [252]. Mice administered a high-fat diet containing grape powder rich in polyphenols demonstrated enhanced glucose tolerance compared to those consuming a high-fat diet exclusively. Quercetin, a polyphenol derived from grape powder, reduced the levels of inflammatory markers such as TNF-α, IL-6, and CD11c in the blood and adipose tissue of mice fed a high-fat diet, as well as MCP-1 and IL-1β in human primary adipocytes [253] (Figure 9). In addition, tea polyphenols suppressed the release of inflammatory cytokines and hepatic fat accumulation tied to obesity in canines on a high-fat feeding plan [254]. 

This research underscores the promising capacity of polyphenols in alleviating adipose tissue inflammation associated with obesity. In conclusion, polyphenols play a crucial role in immunomodulation, obesity prevention, and overall human health. They combat obesity through various pathways, with the most studied being those involving brain neurohormones that regulate insulin-related hunger and satiety signals. In animal models, polyphenols such as resveratrol and curcumin have been shown to reduce hyperinsulinemia, hyperglycemia, inflammation, and cancer, suggesting their potential to prevent and aid in the treatment of obesity. Polyphenols also inhibit fat synthesis and storage by blocking the lipogenic pathway and pro-obesity enzymes like pancreatic lipase. Furthermore, polyphenols enhance thermogenesis, aiding in weight regulation and calorie burning. They influence gut immunity and health by impacting mucosal immunity and inflammation. Polyphenols have effects on T-cells, macrophages, and NK cells, increasing anti-inflammatory cytokines, reducing pro-inflammatory ones, and enhancing NK cell function, thus helping to avoid inflammation-related disorders. Based on multiple studies, it can be concluded that polyphenols may improve health by reducing obesity, immune system alterations, and inflammation. However, further research is necessary to fully understand how polyphenols and their derivatives enhance the immune system, reduce adipose tissue inflammation, and modulate obesity, paving the way for the development of personalized treatments for humans.

## 3. Preclinical and Clinical Data on Polyphenols

Polyphenols are a promising therapeutic agent, and their use in preclinical and clinical research to investigate chronic disease prevention and health benefits has increased [255,256]. Varieties of polyphenols have shown therapeutic potential, but most of the existing research has used preclinical models (Table 1). 

Furthermore, combination chemotherapy with polyphenols, which work through several molecular pathways, has so far been shown to be effective in preclinical studies on various cancer lines and experimental animal models (Table 2). 

Genetic and metabolic differences limit the application of cellular and animal model findings to humans. Thus, polyphenol bioactivities in humans must be thoroughly studied. Some of the already-available clinical data are summarized below (Table 3). Future research will primarily focus on investigating the pharmacokinetics, pharmacodynamics, safety, and mechanisms of action of these medications, including both their therapeutic effects and any potential adverse effects. Below are the results of multiple phytochemical screenings and current clinical trials on polyphenols and polyphenol-based therapies for various disorders.

## 4. Future Perspectives and Takeaway Message

**Figure 10:**
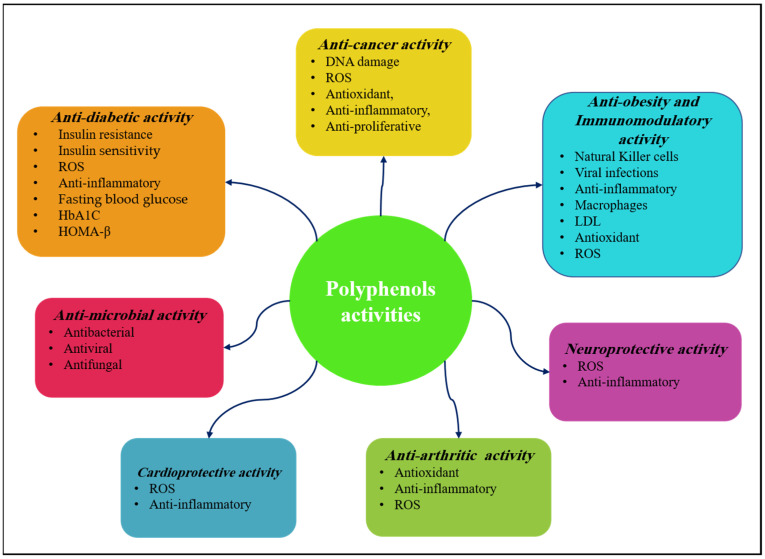
A review of the presently known actions of polyphenols and their potential protective mechanisms based on the available literature.

This article provides a thorough examination of the role of polyphenols in various areas of nutrition research and their possible uses, including for treating obesity, T2D, neurodegenerative diseases, and cancer. Over the past few decades, there has been a significant focus in the scientific literature on polyphenols, and numerous possible health benefits have been revealed (Figure 10). It should be noted that sexual dimorphism plays a crucial role in the aging process, affecting life expectancy. The oxidative–inflammatory theory of aging suggests that aging results from oxidative and inflammatory stress, which cause damage and loss of function in organisms [301]. 

Gender differences in oxidative and inflammatory markers may explain the differences in lifespan between sexes, with males generally exhibiting higher levels of oxidation and basal inflammation. Circulating cell-free DNA, a significant marker of oxidative damage and inflammation, links these two processes and could become a useful marker for aging. Polyphenols, known for their antioxidant properties, are potent in preventing or reducing the harmful effects of various health-related issues. Understanding how oxidative and inflammatory changes differ with aging in each sex is crucial, as it may impact the differences in lifespan observed between the sexes [301]. Abundant evidence suggests that some polyphenols have a beneficial effect on health, specifically in terms of avoiding and delaying specific chronic diseases. The ability to harness these benefits is restricted by the current limited understanding of their mechanisms, the dosage requirements, and their potential adverse effects. It is imperative to analyze the potential negative effects on certain metabolites and carry out more human-based research to confirm the biological mechanisms and public health implications of polyphenols. Prior to performing human supplementation experiments with these metabolites, researchers should take meticulous care to achieve a comprehensive grasp of the mechanisms and implications involved. Experiments conducted in laboratory settings and on animals have employed substantially higher doses of polyphenols than those that are typically seen in the human diet and are safe for their usage. Hence, the precise and advantageous threshold for polyphenol consumption in humans remains uncertain. The study of polyphenols is complex because of the diverse range of molecular structures and the limited knowledge about their absorption in the body. Furthermore, there is a lack of adequate techniques available for quantifying oxidative damage within a living organism, and the assessment of definitive outcomes continues to be difficult. There is a requirement to improve analytical techniques to obtain further data on the processes of absorption and excretion. There is also limited knowledge regarding the long-term impacts of polyphenol intake. Many reports have highlighted the necessity of conducting molecular docking studies to find the prospective polyphenol compounds that may be used to postpone the severity of many undesirable human health disorders. The study of the interactions between polyphenols and receptor molecules in the delay and prevention of acute and chronic diseases is a crucial field for future research. In addition, inadequate stability, low solubility, and restricted bioavailability greatly hinder the application of these metabolites in the fields of food and medicine. Although plant polyphenol metabolites have numerous positive benefits, it has been observed that at high doses, some of these metabolites can exhibit mutagenesis properties and toxicity. Further comprehensive studies on their safety and pharmacological research are necessary to validate the contentious results obtained from sub-chronic and oral toxicity trials. Additional research is necessary to uncover novel polyphenols derived from natural sources to replace the utilization of detrimental synthetic medications. Polyphenols are exposed to a wide variety of chemical substances in their natural habitats, and their abundance of active functional groups enables them to interact with both reactive oxygen species and free radicals. Some of these interactions can be very harmful to human health, making it important to highlight them. The therapeutic effects of drugs are influenced by many factors; among the most significant are the interactions between polyphenols and drug components (such as iron-containing preparations used to treat anemia) and the influence of polyphenols on drug metabolism and pharmacokinetics [302]. Many people understand that certain products can negatively interact with each other; for example, they are aware that certain medications should not be combined with grapefruit juice or herbal infusions. However, many do not fully understand the reasoning behind such advisories. The effects of polyphenols on the activity of drug-metabolizing enzymes underpin this process. These enzymes include phase I and phase II enzymes, such as cytochrome P450, glutathione S-transferase, UDP–glucuronosyltransferase, sulfotransferase, N-acetyltransferase, methyltransferase, epoxide hydrolase, and NAD(P)H–quinone oxidoreductase. To avoid these side effects, both patients and doctors should be aware of the known interactions between the most commonly used drugs and various kinds of food or polyphenolic preparations [190,303,304,305]. Research and development activities are necessary in this area to study the pharmacological effects and ensure a positive and secure future perspective. At present, it is advised to consume fruits, vegetables, and beverages that contain polyphenols. However, it is premature to provide definitive guidelines on the recommended daily intake of polyphenols. Regulatory organizations should give priority to staying informed about scientific findings in order to establish guidelines for the use and supplementation of polyphenols. This encompasses the regulation of health and functional assertions linked to polyphenols, as well as the establishment of dietary guidelines for commonly ingested polyphenols or those that may pose a risk. To promote the adoption of good dietary habits, it is recommended to incorporate recommendations for their consumption into existing nutrition education campaigns and standards. Although there are still gaps in our understanding of this new field, it is essential to take proactive measures in public health to ensure the safety and awareness of individuals.

## 5. Conclusions

Several studies have provided evidence for the protective effects of polyphenols in preventing chronic illnesses. Various mechanisms of action have been suggested to explain these protective effects. Significant advancements have also been achieved in assessing the bioavailability of polyphenols. Nevertheless, further human studies are required to establish conclusive evidence about the beneficial effects of polyphenols. The primary source of definitive evidence will predominantly arise from additional clinical and epidemiological investigations. The number of clinical trials published thus far remains limited. When creating novel products that include polyphenols and are intended to have targeted effects on a specific pathology, it is crucial to ensure that they do not pose an elevated risk for additional pathologies. In the near future, it is expected that the most effective polyphenols and optimal levels of consumption will be identified for both the general population and for individuals at risk of acquiring specific diseases. Prior to achieving this objective, it is essential to use the available information before recommending an increase in their usage. This consideration is also crucial for dietary supplements, as promoting them can lead to a significant rise in polyphenol consumption, often beyond the levels typically obtained from a regular diet. Once the optimal levels of polyphenol intake have been determined, it will be feasible to enhance the nutritional value of food through plant breeding or food processing, and to provide accurate dietary guidelines for promoting good health.

## Figures and Tables

**Table 1:** Preclinical experimental data regarding the pharmacological effects of polyphenols.

Polyphenol	In Vitro/In Vivo Model	Regimen	Outcome	Ref.
Proanthocyanidin, catechin, quercetin	HT-29 cells	Methanolic extract	Restored stress-related GSH reduction by polyphenols in intestinal cells.	[257]
Catechins	MKN 28 cells;	Methanolic extract; polyphenol administration by drinking water or gavage	Prevention of oxidative injury in gastric epithelial cells and gastric mucosa.	[258]
	Male Wistar rats			
Flavonoids, phenolic acids	HT-29 cells; CaCo-2 cells	Cider, apple juice	Increased antioxidant capacity; decreased cellular reactive oxygen species; reduced oxidative cell damage.	[259]
Ellagitannins	Liposome model (large unilamellar vesicles, LUVs)	Pomegranate juice	Inhibition of the lipid peroxidation.	[260]
Tannins; anthocyanins	HT-29 cells	Extract juice	Reduced iNOS and COX-2 levels; modulation of the NF-κB signaling pathway.	[261]
Flavonoids	CaCo-2 cells	Ethanolic extract	Reduced NF-κB transactivation and TNFα transcription levels.	[262]
Catechins	BALB/c mice with DSS-induced colitis	Dietary administration in chow diet	Reductions in TNFα and GSH levels.	[263]
Epigallocatechin-3-gallate	C57/BL6 mice with DSS-induced colitis	Administration of polyphenol mix via oral gavage	Reduction in tissue damage and neutrophile accumulation; increased levels of antioxidant enzymes.	[264]
Polyphenols-rich blueberry extract	Mice	Mice were provided with 15.6 mg/kg BW per day for 12 days	Inhibited body-weight gain and reverted lipid metabolism to normal.	[265]
Rambutan seed extract (containing alkaloids, terpenoids, triterpenoids, and flavonoids)	3T3-L1 cell line	Cells were treated with varying concentrations of the extracts (10 and 50 µg/mL)	Decreased triglyceride levels. Inhibited glucose-6-phosphate dehydrogenase (G6PDH), which promotes adipogenesis.	[266]
Procyanidin-rich grape seed extract (GSPE)	3T3-L1	Cells were treated with 140 mg/L GSPE (dissolved in water) for 24 h on days 0, 2, or 4	Reduced triglyceride content by 32% in cells treated at day zero. Downregulated genes responsible for preadipocyte differentiation but elevated preadipocyte factor-1 (Pref-1).	[267]
Polyphenol-rich cranberry extract	Mice	Mice were provided with 0.75% (*w*/*w*) of a polyphenol-rich cranberry extract per day for 16 weeks	Elevated energy expenditure and brown adipose tissue thermogenesis.	[268]
Polyphenol-rich totum-63 extract	Mice	Mice were fed with a high-fat diet for 12 weeks, followed by supplementation with Totum-63 (2.7% *w*/*w*) for 4 weeks	Decreased body weight and fat mass. Increased expression of insulin receptor β and insulin-induced phosphorylation of PKB in skeletal muscle, white adipose tissue (WAT), and brown adipose tissue (BAT), thereby inducing thermogenesis.	[269]

**Table 2:** Preclinical experimental data regarding the pharmacological effects of polyphenols as an adjuvant to chemotherapy (both in vitro and in vivo).

Polyphenol	Cancer Type	Chemotherapy	Dosage	Assay Type	Molecular Effect(s)	Study Conclusion	Ref.
Curcumin	Lung cancer	Cisplatin	41 µM curcumin + 30 µM cisplatin for A549 cells; 33 µM curcumin + 7 µM cisplatin for H2170 cells	A549 and H2170 cell lines	Suppression of the self-renewal capability of cancer stem cells.	Synergistic inhibition of NSCLC.	[270]
		Crizotinib	30 µM curcumin + 20 µM crizotinib	A549, H460, H1299, and H1066 cell lines	Increased the levels of miR-142-5p through epigenetics and suppressed autophagy.	Enhances NSCLC’s sensitivity to crizotinib treatment.	[271]
	Colorectal cancer	Irinotecan	In vivo: 2–14 μg/mL curcumin + 2–14 ng/mL irinotecan. In vivo: 5 mg/kg curcumin every other day 3 times + irinotecan 25 mg/kg every other day 3 times	CT-26 cell line and C57 BL/6j mice	Upregulated ICD-related proteins including CALR and HMGB1a.	Curcumin may synergistically improve the antitumor effect of irinotecan by promoting the immunogenic cell death (ICD) effect.	[272]
		Oxaliplatin	In vivo: HCT116 and SW480 cells 0–8 µM curcumin + 0.5–32 µM oxaliplatin; HCT116/ oxaliplatin cells 4 µM curcumin + 8 µM oxaliplatin. In vivo: 60 mg/kg curcumin + 10 mg/kg oxaliplatin	HCT116, SW480, and HCT116/oxaliplatin drug-resistant cell lines and BALB/c nude mice	Inhibition of TGF-β/Smad2/Smad3 signaling.	Inhibition of cell proliferation and reduced tumor weight and volume.	[273]
	Liver cancer	5-fluorouracil	In vivo: 5, 10 μM curcumin + 2.5, 5, 10 μM 5-fluorouracil. In vivo: 56.65 mg/kg curcumin + 10 mg/kg 5-fluorouracil	SMMC-7721, Bel-7402, HepG-2, and MHCC97H cell lines and BALB/c nude mice	Decreased expression of NF-κB protein in the nucleus. Increased expression of NF-κB protein in cytoplasm. Downregulation of COX-2 expression.	Synergistic effects and in vivo tumor growth inhibition.	[274]
		Sorafenib	60, 120 μM curcumin + 0.25–10 μM sorafenib	Hep3b and HepG2 cell lines	S-phase and G2/M-phase arrest of liver cancer cells; induced apoptosis; reduced the protein levels of cyclins A, B2, and D1; phosphorylated retinoblastoma and B-cell lymphoma; increased the protein levels of BCL2-associated X protein; cleaved caspase-3; and cleaved caspase-9.	Curcumin augmented the apoptosis-inducing potential of sorafenib.	[275]
	Lung cancer	Crizotinib	30 μM curcumin and 20 μM crizotinib	A549, H460, H1299, and H1066 cells	Increased the levels of miR-142-5p through epigenetics and suppressed autophagy.	Curcumin enhanced NSCLC’s sensitivity to crizotinib treatment.	[271]
Quercetin	Breast cancer	Doxorubicin	0.7 μM quercetin + 2 μg/mL doxorubicin	MCF-10A, MCF-7, and MDA-MB-231 cell lines	Increased the intracellular accumulation of doxorubicin.	Quercetin enhanced doxorubicin apoptotic potential on cancerous cells	[276]
		Lonidamine	80 μM quercetin + 0.1, 1, 5 μM lonidamine	MCF-7 cell line	Induced cell cycle arrest in the G2/M phase; arrested the cell cycle at S point; induced apoptosis through increased caspase levels; decreased MMP-2/-9 mRNA expression.	Synergistic effects.	[277]
	Gastric cancer	5-fluorouracil and doxorubicin	50 μM quercetin + 25 μM 5-fluorouracil; 50 μM quercetin + 0.5 μM doxorubicin	AGS-cyr61 cell line	Reversed multidrug resistance; decreased CYR61, MRP1, and p65; induced caspase-dependent apoptosis; suppressed the migration and downregulation of EMT-related proteins; inhibited colony formations.	Strong synergistic effects with 5-fluorouracil and doxorubicin.	[278]
		Doxorubicin	100–200 μM quercetin+ 0.25–1.25 μM doxorubicin	KATO III cell line	Enhanced apoptosis; induced upregulation of γH2As.	Increases chemotherapeutic effects.	[279]
	Colorectal cancer	Doxorubicin	33 µM quercetin + 0.5 µM doxorubicin	SW620/DOX drug-resistant cell line and SW620/Ad300 cell line	Reversed P-gp-mediated drug resistance; increased intracellular doxorubicin accumulation; modulated glutamine metabolism in doxorubicin-resistant cells via inhibition of SLC1A5.	Reversed multidrug resistance, enhanced sensitivity to doxorubicin.	[280]
		5-fluorouracil	180 µg/mL quercetin + 110 µg/mL 5-fluorouracil	HT-29 cell line	Decreased angiogenesis via inhibition of VEGF.	Synergistically enhanced the anticancer effect of 5-fluorouracil.	[281]
	Breast cancer	Doxorubicin and cisplatin	20 μM quercetin + (0.5 μg/mL doxorubicin + 40 μg/mL cisplatin); 1–40 μM quercetin + (0.5 μg/mL doxorubicin + 40 μg/mL cisplatin)	MDA-MB-231 cell lines	Reduced cardiotoxicity by activating the ERK1/2 pathway in cardiomyocytes; enhanced the antitumor activity of doxorubicin–cisplatin by inhibiting the ERK1/2 pathway in triple-negative breast cancer cells	Enhances the chemotherapeutic effects of doxorubicin–cisplatin; decreases doxorubicin–cisplatin-induced cardiotoxicity.	[282]
Resveratrol	Breast cancer	Cisplatin	12.5, 25, 50 μM resveratrol + 4 μM cisplatin	MDA-MB-231 cell lines and female BALB/c mice MDA-MB-231 xenografts	The expressions of P-AKT, P-PI3K, Smad2, Smad3, P-JNK, and P-ERK induced by TGF-β1 were reversed after resveratrol and cisplatin co-treatment.	Synergistic effect on the inhibition of breast cancer cell viability, migration, and invasion in vivo; enhanced anti-tumor effects and reduced side effects of cisplatin in vivo.	[283]
		Doxorubicin	100, 200, 300 μM resveratrol + 2 mg/mL doxorubicin	MCF-7/ADR drug-resistant cell line	Activation of caspase-8 and caspase-9, inhibition of proliferation and decreased cell viability, miRNA miR-122-5p upregulation and miR-542-3p downregulation, and significantly reduced expression levels of targeted proteins of these miRNAs.	RES chemotherapy sensitizes drug-resistant cancer cell lines.	[284]
	Lung cancer	Gemcitabine	In vivo: 10 µM resveratrol + 1 µM gemcitabine. In vivo: 25 mg/kg gemcitabine i.p. 2×/week + 1 µmol/kg resveratrol 5×/week	HCC827 cell lines and HCC827 xenografts in nude mice	Downregulation of mRNA and the protein levels of ENG; activation of the ERK signaling pathway.	Resveratrol promoted tumor microvessel growth, increased blood perfusion, and promoted drug delivery into tumors, which resulted in an enhanced anticancer effect of gemcitabine.	[285]
	Breast cancer	Paclitaxel	1 μM resveratrol + 1, 10, 100 nM paclitaxel	MCF-7, T47D (ERα+) and MDA-MB 231 (ERα−) cell lines [ER = estrogen receptor]	Decreased neuroglobin levels via interference with the E2/Erα pathway.	Resveratrol increased the sensitivity of cancer cells to paclitaxel and reduced the required dosage of paclitaxel.	[286]

**Table 3:** Overview of clinical trial results regarding the pharmacological efficacy of polyphenols.

Polyphenol/Polyphenol-Based Metabolite	Number of Patients	Dose	Duration	Outcomes	Ref.
Hesperidin	24	500 mg/day	21 days	Enhanced flow-mediated dilatation and decreased levels of inflammatory biomarkers in the blood.	[287]
	100	Daflon 500 mg tablets	7 days	Inflammation, congestion, edema, prolapse, severity, and duration of hemorrhoidal episodes lessened along with their clinical severity.	[288]
	56	379 mg of green-tea extract	90 days	Patients with obesity-related hypertension showed improvements in their blood pressure, insulin resistance, inflammation, oxidative stress, and lipid profile.	[289]
Quercetin	50	500 mg/day	56 days	Improvements in clinical symptoms, disease activity, and hs-TNFα.	[290]
	56	Formulation of nano-hydrogel (0.2%) embedded with quercetin and oleic acid (equimolar doses)	30 days	Reduced wound-healing time and decreased levels of inflammation in diabetic patients.	[291]
Apigenin	100	2 mL of an oleogel preparation of reformulated traditional chamomile oil	Topical application (once)	Pain, nausea, and vomiting significantly decreased in patients with migraines.	[292]
Silymarin (Livergol, Goldaruo Pharmaceutical, Iran)	44	420 mg/day	90 days	Joint swelling, tenderness, and pain were reduced.	[293]
Pycnogenol (Horphag Research Ltd., UK, Geneve, Switzerland)	67	220 mg/day	21 days	C-reactive protein levels and the need for pain relievers and NSAIDs were both reduced in OA patients.	[294]
	100	150 mg/day	90 days	Pain and stiffness were all reduced in OA patients on a daily basis.	[295]
	100	150 mg/day	90 days	OA symptom relief and decreased use of nonsteroidal anti-inflammatory drugs (NSAIDs) and COX-2 inhibitors.	[296]
Alvocidib or Flavopiridol (Tolero Pharmaceuticals, Inc., Salt Lake City, UT, USA)	10	30 min loading dose of 30 mg/m	21 days or 35 days	Patients with chronic lymphocytic leukemia showed less tumor growth.	[297]
Resveratrol	70	20 mg/day	25 days	Decreased dry weights of lipid and cholesterol in removed plaques.	[298]
	18	60 mg/day	28 days	Decreased diastolic blood pressure.	[299]
	62	250 mg/day	90 days	Decrease in hemoglobin A1c, systolic blood pressure, and total cholesterol.	[300]

## References

[1] Guo Q., Li F., Duan Y., Wen C., Wang W., Zhang L., Huang R., Yin Y. (2020). Oxidative Stress, Nutritional Antioxidants and Beyond. Sci. China Life Sci..

[2] Pintea A., Dulf F.V., Bunea A., Socaci S.A., Pop E.A., Opriță V.-A., Giuffrida D., Cacciola F., Bartolomeo G., Mondello L. (2020). Carotenoids, Fatty Acids, and Volatile Compounds in Apricot Cultivars from Romania-A Chemometric Approach. Antioxidants.

[3] Bié J., Sepodes B., Fernandes P.C.B., Ribeiro M.H.L. (2023). Polyphenols in Health and Disease: Gut Microbiota, Bioaccessibility, and Bioavailability. Compounds.

[4] Shamsudin N.F., Ahmed Q.U., Mahmood S., Shah S.A.A., Sarian M.N., Khattak M.M.A.K., Khatib A., Sabere A.S.M., Yusoff Y.M., Latip J. (2022). Flavonoids as Antidiabetic and Anti-Inflammatory Agents: A Review on Structural Activity Relationship-Based Studies and Meta-Analysis. Int. J. Mol. Sci..

[5] Pereira L., Cotas J. (2023). Therapeutic Potential of Polyphenols and Other Micronutrients of Marine Origin. Mar. Drugs.

[6] Pop O.L., Suharoschi R., Socaci S.A., Berger Ceresino E., Weber A., Gruber-Traub C., Vodnar D.C., Fărcaș A.C., Johansson E. (2023). Polyphenols—Ensured Accessibility from Food to the Human Metabolism by Chemical and Biotechnological Treatments. Antioxidants.

[7] Xu L., Zhang X. (2023). Editorial: Dietary Polyphenols, Gut Microbiota, and Human Health. Front. Pharmacol..

[8] Wang S., Du Q., Meng X., Zhang Y. (2022). Natural Polyphenols: A Potential Prevention and Treatment Strategy for Metabolic Syndrome. Food Funct..

[9] Bobrysheva T.N., Anisimov G.S., Zolotoreva M.S., Bobryshev D.V., Budkevich R.O., Moskalev A.A. (2023). Polyphenols as Promising Bioactive Compounds. Probl. Nutr..

[10] Rathod N.B., Elabed N., Punia S., Ozogul F., Kim S.-K., Rocha J.M. (2023). Recent Developments in Polyphenol Applications on Human Health: A Review with Current Knowledge. Plants.

[11] Rasmi Y., Da Silva A.P.G., Rezaei S., Rafique S., Ahmed M.Z. (2022). Biochemical, Molecular, Pharmacokinetic, and Toxicological Aspects of Dietary Polyphenols. Dietary Polyphenols in Human Diseases.

[12] Kabir E.R., Chowdhury N.M., Yasmin H., Kabir T., Akter R., Perveen A., Ashraf G.M., Akter S., Rahman H., Sweilam S.H. (2023). Unveiling the Potential of Polyphenols as Anti-Amyloid Molecules in Alzheimer’sDisease. Curr. Neuropharmacol..

[13] Caponio G.R., Lippolis T., Tutino V., Gigante I., De Nunzio V., Milella R.A., Gasparro M., Notarnicola M. (2022). Nutraceuticals: Focus on Anti-Inflammatory, Anti-Cancer, Antioxidant Properties in Gastrointestinal Tract. Antioxidants.

[14] Dinu M., Tristan Asensi M., Pagliai G., Lotti S., Martini D., Colombini B., Sofi F. (2022). Consumption of Ultra-Processed Foods Is Inversely Associated with Adherence to the Mediterranean Diet: A Cross-Sectional Study. Nutrients.

[15] Negrati M., Razza C., Biasini C., Di Nunzio C., Vancini A., Dall’Asta M., Lovotti G., Trevisi E., Rossi F., Cavanna L. (2021). Mediterranean Diet Affects Blood Circulating Lipid-Soluble Micronutrients and Inflammatory Biomarkers in a Cohort of Breast Cancer Survivors: Results from the SETA Study. Nutrients.

[16] Chen D., Mubeen B., Hasnain A., Rizwan M., Adrees M., Naqvi S.A.H., Iqbal S., Kamran M., El-Sabrout A.M., Elansary H.O. (2022). Role of Promising Secondary Metabolites to Confer Resistance Against Environmental Stresses in Crop Plants: Current Scenario and Future Perspectives. Front. Plant Sci..

[17] Eseberri I., Trepiana J., Léniz A., Gómez-García I., Carr-Ugarte H., González M., Portillo M.P. (2022). Variability in the Beneficial Effects of Phenolic Compounds: A Review. Nutrients.

[18] Grgić J., Šelo G., Planinić M., Tišma M., Bucić-Kojić A. (2020). Role of the Encapsulation in Bioavailability of Phenolic Compounds. Antioxidants.

[19] Murakami A. (2024). Impact of Hormesis to Deepen Our Understanding of the Mechanisms Underlying the Bioactivities of Polyphenols. Curr. Opin. Biotechnol..

[20] Melrose J. (2023). The Potential of Flavonoids and Flavonoid Metabolites in the Treatment of Neurodegenerative Pathology in Disorders of Cognitive Decline. Antioxidants.

[21] Xie Y., Gong T., Liu H., Fan Z., Zhaojun C., Liu X. (2022). In Vitro and In Vivo Digestive Fate and Antioxidant Activities of Polyphenols from Hulless Barley: Impact of Various Thermal Processing Methods and β-Glucan. J. Agric. Food Chem..

[22] Farhan M., Rizvi A. (2023). The Pharmacological Properties of Red Grape Polyphenol Resveratrol: Clinical Trials and Obstacles in Drug Development. Nutrients.

[23] Aatif M. (2023). Current Understanding of Polyphenols to Enhance Bioavailability for Better Therapies. Biomedicines.

[24] Szczepańska P., Rychlicka M., Groborz S., Kruszyńska A., Ledesma-Amaro R., Rapak A., Gliszczyńska A., Lazar Z. (2023). Studies on the Anticancer and Antioxidant Activities of Resveratrol and Long-Chain Fatty Acid Esters. Int. J. Mol. Sci..

[25] Patel H., Li J., Bo L., Mehta R., Ashby C.R., Wang S., Cai W., Chen Z.-S. (2024). Nanotechnology-Based Delivery Systems to Overcome Drug Resistance in Cancer. Med. Rev..

[26] Sang S. (2022). Impacts of Biotransformation on the Health Benefits of Tea Polyphenols. J. Nutr. Sci. Vitaminol..

[27] Sahadevan R., Singh S., Binoy A., Sadhukhan S. (2023). Chemico-Biological Aspects of (-)-Epigallocatechin-3-Gallate (EGCG) to Improve Its Stability, Bioavailability and Membrane Permeability: Current Status and Future Prospects. Crit. Rev. Food Sci. Nutr..

[28] Gonzales G.B., Smagghe G., Grootaert C., Zotti M., Raes K., Van Camp J. (2015). Flavonoid Interactions during Digestion, Absorption, Distribution and Metabolism: A Sequential Structure-Activity/Property Relationship-Based Approach in the Study of Bioavailability and Bioactivity. Drug Metab. Rev..

[29] Wang Q., Yu Q., Wu M. (2022). Antioxidant and Neuroprotective Actions of Resveratrol in Cerebrovascular Diseases. Front. Pharmacol..

[30] Gadacha W., Ben-Attia M., Bonnefont-Rousselot D., Aouani E., Ghanem-Boughanmi N., Touitou Y. (2009). Resveratrol Opposite Effects on Rat Tissue Lipoperoxidation: Pro-Oxidant during Day-Time and Antioxidant at Night. Redox Rep..

[31] Zhang W., Qi S., Xue X., Al Naggar Y., Wu L., Wang K. (2021). Understanding the Gastrointestinal Protective Effects of Polyphenols Using Foodomics-Based Approaches. Front. Immunol..

[32] Walle T. (2009). Methylation of Dietary Flavones Increases Their Metabolic Stability and Chemopreventive Effects. Int. J. Mol. Sci..

[33] Luca S.V., Macovei I., Bujor A., Miron A., Skalicka-Woźniak K., Aprotosoaie A.C., Trifan A. (2020). Bioactivity of Dietary Polyphenols: The Role of Metabolites. Crit. Rev. Food Sci. Nutr..

[34] Simões R., Ribeiro A.C., Dias R., Freitas V., Soares S., Pérez-Gregorio R. (2024). Unveiling the Immunomodulatory Potential of Phenolic Compounds in Food Allergies. Nutrients.

[35] Mahajan R., Attri S., Mehta V., Udayabanu M., Goel G. (2018). Microbe-Bio-Chemical Insight: Reviewing Interactions between Dietary Polyphenols and Gut Microbiota. Mini Rev. Med. Chem..

[36] Lessard-Lord J., Roussel C., Lupien-Meilleur J., Généreux P., Richard V., Guay V., Roy D., Desjardins Y. (2024). Short Term Supplementation with Cranberry Extract Modulates Gut Microbiota in Human and Displays a Bifidogenic Effect. NPJ Biofilms Microbiomes.

[37] Teng H., Chen L. (2019). Polyphenols and Bioavailability: An Update. Crit. Rev. Food Sci. Nutr..

[38] Di Lorenzo C., Colombo F., Biella S., Stockley C., Restani P. (2021). Polyphenols and Human Health: The Role of Bioavailability. Nutrients.

[39] Anand S., Sowbhagya R., Ansari M.A., Alzohairy M.A., Alomary M.N., Almalik A.I., Ahmad W., Tripathi T., Elderdery A.Y. (2022). Polyphenols and Their Nanoformulations: Protective Effects against Human Diseases. Life.

[40] Rajput A., Sharma P., Singh D., Singh S., Kaur P., Attri S., Mohana P., Kaur H., Rashid F., Bhatia A. (2023). Role of Polyphenolic Compounds and Their Nanoformulations: A Comprehensive Review on Cross-Talk between Chronic Kidney and Cardiovascular Diseases. Naunyn Schmiedebergs Arch. Pharmacol..

[41] Page M.J., McKenzie J.E., Bossuyt P.M., Boutron I., Hoffmann T.C., Mulrow C.D., Shamseer L., Tetzlaff J.M., Akl E.A., Brennan S.E. (2021). The PRISMA 2020 Statement: An Updated Guideline for Reporting Systematic Reviews. BMJ.

[42] Farhan M. (2024). The Promising Role of Polyphenols in Skin Disorders. Molecules.

[43] Purgatorio R., Boccarelli A., Pisani L., De Candia M., Catto M., Altomare C. (2024). A Critical Appraisal of the Protective Activity of Polyphenolic Antioxidants against Iatrogenic Effects of Anticancer Chemotherapeutics. Antioxidants.

[44] Patra S., Pradhan B., Nayak R., Behera C., Das S., Patra S.K., Efferth T., Jena M., Bhutia S.K. (2021). Dietary Polyphenols in Chemoprevention and Synergistic Effect in Cancer: Clinical Evidences and Molecular Mechanisms of Action. Phytomedicine.

[45] Yi J., Li S., Wang C., Cao N., Qu H., Cheng C., Wang Z., Wang L., Zhou L. (2019). Potential Applications of Polyphenols on Main ncRNAs Regulations as Novel Therapeutic Strategy for Cancer. Biomed. Pharmacother..

[46] Symonds E.L., Konczak I., Fenech M. (2013). The Australian Fruit Illawarra Plum (*Podocarpus elatus* Endl., Podocarpaceae) Inhibits Telomerase, Increases Histone Deacetylase Activity and Decreases Proliferation of Colon Cancer Cells. Br. J. Nutr..

[47] Pandareesh M.D., Mythri R.B., Srinivas Bharath M.M. (2015). Bioavailability of Dietary Polyphenols: Factors Contributing to Their Clinical Application in CNS Diseases. Neurochem. Int..

[48] Amawi H., Ashby C., Samuel T., Peraman R., Tiwari A. (2017). Polyphenolic Nutrients in Cancer Chemoprevention and Metastasis: Role of the Epithelial-to-Mesenchymal (EMT) Pathway. Nutrients.

[49] Bhosale P.B., Ha S.E., Vetrivel P., Kim H.H., Kim S.M., Kim G.S. (2020). Functions of Polyphenols and Its Anticancer Properties in Biomedical Research: A Narrative Review. Transl. Cancer Res. TCR.

[50] Majidinia M., Bishayee A., Yousefi B. (2019). Polyphenols: Major Regulators of Key Components of DNA Damage Response in Cancer. DNA Repair..

[51] Ahire V., Kumar A., Mishra K.P., Kulkarni G. (2017). Ellagic Acid Enhances Apoptotic Sensitivity of Breast Cancer Cells to γ-Radiation. Nutr. Cancer.

[52] Hashemi Sheikhshabani S., Amini-Farsani Z., Rahmati S., Jazaeri A., Mohammadi-Samani M., Asgharzade S. (2021). Oleuropein Reduces Cisplatin Resistance in Ovarian Cancer by Targeting Apoptotic Pathway Regulators. Life Sci..

[53] Chen C., Ai Q., Wei Y. (2021). Hydroxytyrosol Protects against Cisplatin-Induced Nephrotoxicity via Attenuating CKLF1 Mediated Inflammation, and Inhibiting Oxidative Stress and Apoptosis. Int. Immunopharmacol..

[54] Badolato M., Carullo G., Cione E., Aiello F., Caroleo M.C. (2017). From the Hive: Honey, a Novel Weapon against Cancer. Eur. J. Med. Chem..

[55] Attia W.Y., Gabry M.S., El-Shaikh K.A., Othman G.A. (2008). The Anti-Tumor Effect of Bee Honey in Ehrlich Ascite Tumor Model of Mice Is Coincided with Stimulation of the Immune Cells. Egypt. J. Immunol..

[56] Takruri H.R., Shomaf M.S., Shnaigat S.F. (2017). Multi Floral Honey Has a Protective Effect against Mammary Cancer Induced by 7,12-Dimethylbenz(a)Anthracene in Sprague Dawley Rats. JAS.

[57] Raeessi M.A., Raeessi N., Panahi Y., Gharaie H., Davoudi S.M., Saadat A., Karimi Zarchi A.A., Raeessi F., Ahmadi S.M., Jalalian H. (2014). “Coffee plus Honey” versus “Topical Steroid” in the Treatment of Chemotherapy-Induced Oral Mucositis: A Randomised Controlled Trial. BMC Complement. Altern. Med..

[58] Charalambous A., Lambrinou E., Katodritis N., Vomvas D., Raftopoulos V., Georgiou M., Paikousis L., Charalambous M. (2017). The Effectiveness of Thyme Honey for the Management of Treatment-Induced Xerostomia in Head and Neck Cancer Patients: A Feasibility Randomized Control Trial. Eur. J. Oncol. Nurs..

[59] Neamatallah T., El-Shitany N.A., Abbas A.T., Ali S.S., Eid B.G. (2018). Honey Protects against Cisplatin-Induced Hepatic and Renal Toxicity through Inhibition of NF-κB-Mediated COX-2 Expression and the Oxidative Stress Dependent BAX/Bcl-2/Caspase-3 Apoptotic Pathway. Food Funct..

[60] AronPharma Sp. z o. o (2023). Polyphenol Rich Aerosol as a Support for Cancer Patients in Minimizing Side Effects After a Radiation Therapy.

[61] AronPharma Sp. z o. o (2023). Investigation of a Polyphenol-Rich Preparation as Support for Oncology Patients Undergoing Gastrointestinal Tumor Resection.

[62] Xue D., Peng Y., Zhang M., Zheng L., Liang Q., Li H., Yu J.-S., Chen J.-T. (2020). Compositions and Methods for Preventing and Treating Radiation-Induced Bystander Effects Caused by Radiation or Radiotherapy.

[63] Donati M.B. (2021). Supplementation With Dietary Anthocyanins and Side Effects of Radiotherapy for Breast Cancer. https://clinicaltrials.gov/study/NCT02195960.

[64] Orchard T. (2023). Protecting the Brain From Toxic Side Effects of Chemotherapy: A Pilot Study of a MIND Diet Intervention in Women Undergoing Active Treatment for Breast Cancer.

[65] Arabshomali A., Bazzazzadehgan S., Mahdi F., Shariat-Madar Z. (2023). Potential Benefits of Antioxidant Phytochemicals in Type 2 Diabetes. Molecules.

[66] Nyakundi B.B., Yang J. (2023). Uses of Papaya Leaf and Seaweed Supplementations for Controlling Glucose Homeostasis in Diabetes. Int. J. Mol. Sci..

[67] Han X., Wu Y.-C., Meng M., Sun Q.-S., Gao S.-M., Sun H. (2018). Linarin Prevents LPS-induced Acute Lung Injury by Suppressing Oxidative Stress and Inflammation via Inhibition of TXNIP/NLRP3 and NF-κB Pathways. Int. J. Mol. Med..

[68] Wang T., Shan M.-Y., Tang C.-Y., Cheng M.-Y., Chen B., Yan J., Xu Z.-H. (2023). Linarin Ameliorates Diabetic Liver Injury by Alleviating Oxidative Stress and Inflammation through the Inhibition of AKR1B1. Comb. Chem. High. Throughput Screen..

[69] Lee J., Mitchell A.E. (2011). Quercetin and Isorhamnetin Glycosides in Onion (*Allium cepa* L.): Varietal Comparison, Physical Distribution, Coproduct Evaluation, and Long-Term Storage Stability. J. Agric. Food Chem..

[70] Abdel Motaal A., Salem H.H., Almaghaslah D., Alsayari A., Bin Muhsinah A., Alfaifi M.Y., Elbehairi S.E.I., Shati A.A., El-Askary H. (2020). Flavonol Glycosides: In Vitro Inhibition of DPPIV, Aldose Reductase and Combating Oxidative Stress Are Potential Mechanisms for Mediating the Antidiabetic Activity of Cleome Droserifolia. Molecules.

[71] Kalai F.Z., Boulaaba M., Ferdousi F., Isoda H. (2022). Effects of Isorhamnetin on Diabetes and Its Associated Complications: A Review of In Vitro and In Vivo Studies and a Post Hoc Transcriptome Analysis of Involved Molecular Pathways. Int. J. Mol. Sci..

[72] Zhang X.-F., Tang Y.-J., Guan X.-X., Lu X., Li J., Chen X.-L., Deng J.-L., Fan J.-M. (2022). Flavonoid Constituents of Amomum Tsao-Ko Crevost et Lemarie and Their Antioxidant and Antidiabetic Effects in Diabetic Rats—In Vitro and in Vivo Studies. Food Funct..

[73] Yan X., Qi M., Li P., Zhan Y., Shao H. (2017). Apigenin in Cancer Therapy: Anti-Cancer Effects and Mechanisms of Action. Cell Biosci..

[74] Laaroussi H., Bakour M., Ousaaid D., Aboulghazi A., Ferreira-Santos P., Genisheva Z., Teixeira J.A., Lyoussi B. (2020). Effect of Antioxidant-Rich Propolis and Bee Pollen Extracts against D-Glucose Induced Type 2 Diabetes in Rats. Food Res. Int..

[75] Alam W., Rocca C., Khan H., Hussain Y., Aschner M., De Bartolo A., Amodio N., Angelone T., Cheang W.S. (2021). Current Status and Future Perspectives on Therapeutic Potential of Apigenin: Focus on Metabolic-Syndrome-Dependent Organ Dysfunction. Antioxidants.

[76] Bakour M., Laaroussi H., Ferreira-Santos P., Genisheva Z., Ousaaid D., Teixeira J.A., Lyoussi B. (2022). Exploring the Palynological, Chemical, and Bioactive Properties of Non-Studied Bee Pollen and Honey from Morocco. Molecules.

[77] Luo Z., Fu C., Li T., Gao Q., Miao D., Xu J., Zhao Y. (2021). Hypoglycemic Effects of Licochalcone A on the Streptozotocin-Induced Diabetic Mice and Its Mechanism Study. J. Agric. Food Chem..

[78] Luo Z., Li T., Gao Q., Chen Y., Su G., Zhao Y. (2021). Impact of Licochalcone A on the Progression of Diabetic Nephropathy in Type 2 Diabetes Mellitus of C57BL/6 Mice. Food Funct..

[79] Ahangarpour A., Oroojan A.A., Khorsandi L., Kouchak M., Badavi M. (2018). Solid Lipid Nanoparticles of Myricitrin Have Antioxidant and Antidiabetic Effects on Streptozotocin-Nicotinamide-Induced Diabetic Model and Myotube Cell of Male Mouse. Oxid. Med. Cell Longev..

[80] Oza M.J., Kulkarni Y.A. (2018). Biochanin A Improves Insulin Sensitivity and Controls Hyperglycemia in Type 2 Diabetes. Biomed. Pharmacother..

[81] Amri J., Alaee M., Babaei R., Salemi Z., Meshkani R., Ghazavi A., Akbari A., Salehi M. (2022). Biochanin-A Has Antidiabetic, Antihyperlipidemic, Antioxidant, and Protective Effects on Diabetic Nephropathy via Suppression of TGF-Β1 and PAR-2 Genes Expression in Kidney Tissues of STZ-Induced Diabetic Rats. Biotechnol. Appl. Biochem..

[82] Ram C., Gairola S., Verma S., Mugale M.N., Bonam S.R., Murty U.S., Sahu B.D. (2023). Biochanin A Ameliorates Nephropathy in High-Fat Diet/Streptozotocin-Induced Diabetic Rats: Effects on NF-kB/NLRP3 Axis, Pyroptosis, and Fibrosis. Antioxidants.

[83] Oza M.J., Kulkarni Y.A. (2018). Formononetin Treatment in Type 2 Diabetic Rats Reduces Insulin Resistance and Hyperglycemia. Front. Pharmacol..

[84] Sadri H., Goodarzi M.T., Salemi Z., Seifi M. (2017). Antioxidant Effects of Biochanin A in Streptozotocin Induced Diabetic Rats. Braz. Arch. Biol. Technol..

[85] Mou X., Zhou D.-Y., Zhou D.-Y., Ma J.-R., Liu Y.-H., Chen H.-P., Hu Y.-B., Shou C.-M., Chen J.-W., Liu W.-H. (2016). Serum TGF-Β1 as a Biomarker for Type 2 Diabetic Nephropathy: A Meta-Analysis of Randomized Controlled Trials. PLoS ONE.

[86] Bagang N., Gupta K., Singh G., Kanuri S.H., Mehan S. (2023). Protease-Activated Receptors in Kidney Diseases: A Comprehensive Review of Pathological Roles, Therapeutic Outcomes and Challenges. Chem. Biol. Interact..

[87] Tay K.-C., Tan L.T.-H., Chan C.K., Hong S.L., Chan K.-G., Yap W.H., Pusparajah P., Lee L.-H., Goh B.-H. (2019). Formononetin: A Review of Its Anticancer Potentials and Mechanisms. Front. Pharmacol..

[88] Lossi L. (2022). The Concept of Intrinsic versus Extrinsic Apoptosis. Biochem. J..

[89] Jain P.G., Nayse P.G., Patil D.J., Shinde S.D., Surana S.J. (2020). The Possible Antioxidant Capabilities of Formononetin in Guarding against Streptozotocin-Induced Diabetic Nephropathy in Rats. Future J. Pharm. Sci..

[90] Hou X., Xu S., Maitland-Toolan K.A., Sato K., Jiang B., Ido Y., Lan F., Walsh K., Wierzbicki M., Verbeuren T.J. (2008). SIRT1 Regulates Hepatocyte Lipid Metabolism through Activating AMP-Activated Protein Kinase. J. Biol. Chem..

[91] Zhang L., Keung W., Samokhvalov V., Wang W., Lopaschuk G.D. (2010). Role of Fatty Acid Uptake and Fatty Acid Beta-Oxidation in Mediating Insulin Resistance in Heart and Skeletal Muscle. Biochim. Biophys. Acta.

[92] Zhou Z., Zhou X., Dong Y., Li M., Xu Y. (2019). Formononetin Ameliorates High Glucose-induced Endothelial Dysfunction by Inhibiting the JAK/STAT Signaling Pathway. Mol. Med. Rep..

[93] Lv J., Zhuang K., Jiang X., Huang H., Quan S. (2020). Renoprotective Effect of Formononetin by Suppressing Smad3 Expression in Db/Db Mice. Diabetes Metab. Syndr. Obes..

[94] Constantin R.P., Constantin J., Pagadigorria C.L.S., Ishii-Iwamoto E.L., Bracht A., de Ono M.K.C., Yamamoto N.S. (2010). The Actions of Fisetin on Glucose Metabolism in the Rat Liver. Cell Biochem. Funct..

[95] Prasath G.S., Pillai S.I., Subramanian S.P. (2014). Fisetin Improves Glucose Homeostasis through the Inhibition of Gluconeogenic Enzymes in Hepatic Tissues of Streptozotocin Induced Diabetic Rats. Eur. J. Pharmacol..

[96] Vinayagam R., Xu B. (2015). Antidiabetic Properties of Dietary Flavonoids: A Cellular Mechanism Review. Nutr. Metab..

[97] Li Y., Ding Y. (2012). Minireview: Therapeutic Potential of Myricetin in Diabetes Mellitus. Food Sci. Hum. Wellness.

[98] Lalitha N., Sadashivaiah B., Ramaprasad T.R., Singh S.A. (2020). Anti-Hyperglycemic Activity of Myricetin, through Inhibition of DPP-4 and Enhanced GLP-1 Levels, Is Attenuated by Co-Ingestion with Lectin-Rich Protein. PLoS ONE.

[99] Li Y., Zheng X., Yi X., Liu C., Kong D., Zhang J., Gong M. (2017). Myricetin: A Potent Approach for the Treatment of Type 2 Diabetes as a Natural Class B GPCR Agonist. FASEB J..

[100] Zhao Z., Chen Y., Li X., Zhu L., Wang X., Li L., Sun H., Han X., Li J. (2022). Myricetin Relieves the Symptoms of Type 2 Diabetes Mice and Regulates Intestinal Microflora. Biomed. Pharmacother..

[101] Les F., Cásedas G., Gómez C., Moliner C., Valero M.S., López V. (2021). The Role of Anthocyanins as Antidiabetic Agents: From Molecular Mechanisms to in Vivo and Human Studies. J. Physiol. Biochem..

[102] Cásedas G., Les F., Gómez-Serranillos M.P., Smith C., López V. (2017). Anthocyanin Profile, Antioxidant Activity and Enzyme Inhibiting Properties of Blueberry and Cranberry Juices: A Comparative Study. Food Funct..

[103] Khan D., Sharif A., Zafar M., Akhtar B., Akhtar M.F., Awan S. (2020). Delonix Regia a Folklore Remedy for Diabetes; Attenuates Oxidative Stress and Modulates Type II Diabetes Mellitus. Curr. Pharm. Biotechnol..

[104] Meng Q., Qi X., Fu Y., Chen Q., Cheng P., Yu X., Sun X., Wu J., Li W., Zhang Q. (2020). Flavonoids Extracted from Mulberry (*Morus alba* L.) Leaf Improve Skeletal Muscle Mitochondrial Function by Activating AMPK in Type 2 Diabetes. J. Ethnopharmacol..

[105] Thaipitakwong T., Numhom S., Aramwit P. (2018). Mulberry Leaves and Their Potential Effects against Cardiometabolic Risks: A Review of Chemical Compositions, Biological Properties and Clinical Efficacy. Pharm. Biol..

[106] Thaipitakwong T., Supasyndh O., Rasmi Y., Aramwit P. (2020). A Randomized Controlled Study of Dose-Finding, Efficacy, and Safety of Mulberry Leaves on Glycemic Profiles in Obese Persons with Borderline Diabetes. Complement. Ther. Med..

[107] Uchiyama Y., Suzuki T., Mochizuki K., Goda T. (2013). Dietary Supplementation with (-)-Epigallocatechin-3-Gallate Reduces Inflammatory Response in Adipose Tissue of Non-Obese Type 2 Diabetic Goto-Kakizaki (GK) Rats. J. Agric. Food Chem..

[108] Elbling L., Weiss R.-M., Teufelhofer O., Uhl M., Knasmueller S., Schulte-Hermann R., Berger W., Micksche M. (2005). Green Tea Extract and (-)-Epigallocatechin-3-Gallate, the Major Tea Catechin, Exert Oxidant but Lack Antioxidant Activities. FASEB J..

[109] Ahangarpour A., Afshari G., Mard S.A., Khodadadi A., Hashemitabar M. (2016). Preventive Effects of Procyanidin A2 on Glucose Homeostasis, Pancreatic and Duodenal Homebox 1, and Glucose Transporter 2 Gene Expression Disturbance Induced by Bisphenol A in Male Mice. J. Physiol. Pharmacol..

[110] Wen L., Wu D., Tan X., Zhong M., Xing J., Li W., Li D., Cao F. (2022). The Role of Catechins in Regulating Diabetes: An Update Review. Nutrients.

[111] Draganescu D., Andritoiu C., Hritcu D., Dodi G., Popa M.I. (2021). Flaxseed Lignans and Polyphenols Enhanced Activity in Streptozotocin-Induced Diabetic Rats. Biology.

[112] Jyoti M.A., Shah M.S., Uddin M.N., Hossain M.K., Han A., Geng P., Islam M.N., Mamun A.A. (2024). Anti-Oxidant and Neuro-Modulatory Effects of Bioactive Byttneria Pilosa Leaf Extract in Swiss Albino Mice Using Behavioral Models. Front. Chem..

[113] Paul B.M., Jagadeesan G., Kannan G., Jegan Raj F., Annadurai Y., Piramanayagam S., Thangaraj P. (2024). Exploring the Hypoglycaemic Efficacy of Bio-Accessed Antioxidative Polyphenolics in Thermally Processed Cucumis Dipsaceus Fruits—An in Vitro and in Silico Study. Food Chem..

[114] Sung S., Kwon D., Um E., Kim B. (2019). Could Polyphenols Help in the Control of Rheumatoid Arthritis?. Molecules.

[115] Panche A.N., Diwan A.D., Chandra S.R. (2016). Flavonoids: An Overview. J. Nutr. Sci..

[116] Doss H.M., Samarpita S., Ganesan R., Rasool M. (2018). Ferulic Acid, a Dietary Polyphenol Suppresses Osteoclast Differentiation and Bone Erosion via the Inhibition of RANKL Dependent NF-κB Signalling Pathway. Life Sci..

[117] Kwak S.C., Lee C., Kim J.-Y., Oh H.M., So H.-S., Lee M.S., Rho M.C., Oh J. (2013). Chlorogenic Acid Inhibits Osteoclast Differentiation and Bone Resorption by Down-Regulation of Receptor Activator of Nuclear Factor Kappa-B Ligand-Induced Nuclear Factor of Activated T Cells C1 Expression. Biol. Pharm. Bull..

[118] Neog M.K., Joshua Pragasam S., Krishnan M., Rasool M. (2017). P-Coumaric Acid, a Dietary Polyphenol Ameliorates Inflammation and Curtails Cartilage and Bone Erosion in the Rheumatoid Arthritis Rat Model. Biofactors.

[119] Tsai M.-H., Hsu L.-F., Lee C.-W., Chiang Y.-C., Lee M.-H., How J.-M., Wu C.-M., Huang C.-L., Lee I.-T. (2017). Resveratrol Inhibits Urban Particulate Matter-Induced COX-2/PGE2 Release in Human Fibroblast-like Synoviocytes via the Inhibition of Activation of NADPH Oxidase/ROS/NF-κB. Int. J. Biochem. Cell Biol..

[120] Wahba M.G.F., Messiha B.A.S., Abo-Saif A.A. (2016). Protective Effects of Fenofibrate and Resveratrol in an Aggressive Model of Rheumatoid Arthritis in Rats. Pharm. Biol..

[121] Chen Z., Xiao G., Ao J. (2024). Resveratrol Attenuates Rheumatoid Arthritis Induce Neutrophil Extracellular Traps via TLR-4 Mediated Inflammation in C57BL/6 Mice. Physiol. Res..

[122] Ahmed S., Ahmed K.S., Rahman M.N., Hossain H., Han A., Geng P., Daula A.F.M.S.U., Mamun A.A. (2024). Polyphenols and Extracts from Zingiber Roseum (Roxb.) Roscoe Leaf Mitigate Pain, Inflammation and Pyrexia by Inhibiting Cyclooxygenase-2: An in Vivo and in Silico Studies. Front. Pharmacol..

[123] Sirše M. (2022). Effect of Dietary Polyphenols on Osteoarthritis—Molecular Mechanisms. Life.

[124] Ansari M.Y., Ahmad N., Haqqi T.M. (2020). Oxidative Stress and Inflammation in Osteoarthritis Pathogenesis: Role of Polyphenols. Biomed. Pharmacother..

[125] Li W., Wang Y., Tang Y., Lu H., Qi Y., Li G., He H., Lu F., Yang Y., Sun H. (2021). Quercetin Alleviates Osteoarthritis Progression in Rats by Suppressing Inflammation and Apoptosis via Inhibition of IRAK1/NLRP3 Signaling. J. Inflamm. Res..

[126] Hu Y., Gui Z., Zhou Y., Xia L., Lin K., Xu Y. (2019). Quercetin Alleviates Rat Osteoarthritis by Inhibiting Inflammation and Apoptosis of Chondrocytes, Modulating Synovial Macrophages Polarization to M2 Macrophages. Free Radic. Biol. Med..

[127] Wang X.-P., Xie W.-P., Bi Y.-F., Wang B.-A., Song H.-B., Wang S.-L., Bi R.-X. (2021). Quercetin Suppresses Apoptosis of Chondrocytes Induced by IL-1β via Inactivation of P38 MAPK Signaling Pathway. Exp. Ther. Med..

[128] Heydari Nasrabadi M., Parsivand M., Mohammadi N., Asghari Moghaddam N. (2022). Comparison of *Elaeagnus angustifolia* L. Extract and Quercetin on Mouse Model of Knee Osteoarthritis. J. Ayurveda Integr. Med..

[129] Kuršvietienė L., Stanevičienė I., Mongirdienė A., Bernatonienė J. (2016). Multiplicity of Effects and Health Benefits of Resveratrol. Medicina.

[130] Oliviero F., Zamudio-Cuevas Y., Belluzzi E., Andretto L., Scanu A., Favero M., Ramonda R., Ravagnan G., López-Reyes A., Spinella P. (2019). Polydatin and Resveratrol Inhibit the Inflammatory Process Induced by Urate and Pyrophosphate Crystals in THP-1 Cells. Foods.

[131] Xu X., Liu X., Yang Y., He J., Jiang M., Huang Y., Liu X., Liu L., Gu H. (2020). Resveratrol Exerts Anti-Osteoarthritic Effect by Inhibiting TLR4/NF-κB Signaling Pathway via the TLR4/Akt/FoxO1 Axis in IL-1β-Stimulated SW1353 Cells. Drug Des. Dev. Ther..

[132] Marouf B.H. (2021). Effect of Resveratrol on Serum Levels of Type II Collagen and Aggrecan in Patients with Knee Osteoarthritis: A Pilot Clinical Study. Biomed. Res. Int..

[133] Su C.-Y., Luo Y., Fang C.-H., Fang H.-W. (2021). The Effects of Antioxidant Supplements on the Inflammatory Gene Expression of Osteoarthritis-like Chondrocytes. Appl. Sci..

[134] Long Z., Xiang W., Li J., Yang T., Yu G. (2021). Exploring the Mechanism of Resveratrol in Reducing the Soft Tissue Damage of Osteoarthritis Based on Network Pharmacology and Experimental Pharmacology. Evid. Based Complement. Altern. Med..

[135] Shep D., Khanwelkar C., Gade P., Karad S. (2020). Efficacy and Safety of Combination of Curcuminoid Complex and Diclofenac versus Diclofenac in Knee Osteoarthritis: A Randomized Trial. Medicine.

[136] Nakagawa Y., Mukai S., Yamada S., Murata S., Yabumoto H., Maeda T., Akamatsu S. (2020). The Efficacy and Safety of Highly-Bioavailable Curcumin for Treating Knee Osteoarthritis: A 6-Month Open-Labeled Prospective Study. Clin. Med. Insights Arthritis Musculoskelet. Disord..

[137] Basak S., Hridayanka K.S.N., Duttaroy A.K. (2024). Bioactives and Their Roles in Bone Metabolism of Osteoarthritis: Evidence and Mechanisms on Gut-Bone Axis. Front. Immunol..

[138] Lee H.E., Yang G., Park Y.B., Kang H.C., Cho Y.-Y., Lee H.S., Lee J.Y. (2019). Epigallocatechin-3-Gallate Prevents Acute Gout by Suppressing NLRP3 Inflammasome Activation and Mitochondrial DNA Synthesis. Molecules.

[139] Rasheed Z., Rasheed N., Al-Shaya O. (2018). Epigallocatechin-3-O-Gallate Modulates Global microRNA Expression in Interleukin-1β-Stimulated Human Osteoarthritis Chondrocytes: Potential Role of EGCG on Negative Co-Regulation of microRNA-140-3p and ADAMTS5. Eur. J. Nutr..

[140] Manso T., Lores M., De Miguel T. (2021). Antimicrobial Activity of Polyphenols and Natural Polyphenolic Extracts on Clinical Isolates. Antibiotics.

[141] Betts J.W., Hornsey M., Higgins P.G., Lucassen K., Wille J., Salguero F.J., Seifert H., La Ragione R.M. (2019). Restoring the Activity of the Antibiotic Aztreonam Using the Polyphenol Epigallocatechin Gallate (EGCG) against Multidrug-Resistant Clinical Isolates of Pseudomonas Aeruginosa. J. Med. Microbiol..

[142] Czerkas K., Olchowik-Grabarek E., Łomanowska M., Abdulladjanova N., Sękowski S. (2024). Antibacterial Activity of Plant Polyphenols Belonging to the Tannins against Streptococcus Mutans—Potential against Dental Caries. Molecules.

[143] Bouloumpasi E., Hatzikamari M., Christaki S., Lazaridou A., Chatzopoulou P., Biliaderis C.G., Irakli M. (2024). Assessment of Antioxidant and Antibacterial Potential of Phenolic Extracts from Post-Distillation Solid Residues of Oregano, Rosemary, Sage, Lemon Balm, and Spearmint. Processes.

[144] Bouymajane A., Filali F.R., Moujane S., Majdoub Y.O.E., Otzen P., Channaoui S., Ed-Dra A., Bouddine T., Sellam K., Boughrous A.A. (2024). Phenolic Compound, Antioxidant, Antibacterial, and In Silico Studies of Extracts from the Aerial Parts of *Lactuca saligna* L. Molecules.

[145] Chlif N., Bouymajane A., Oulad El Majdoub Y., Diouri M., Rhazi Filali F., Bentayeb A., Altemimi A.B., Mondello L., Cacciola F. (2022). Phenolic Compounds, in Vivo Anti-Inflammatory, Analgesic and Antipyretic Activities of the Aqueous Extracts from Fresh and Dry Aerial Parts of *Brocchia cinerea* (Vis.). J. Pharm. Biomed. Anal..

[146] Miklasińska-Majdanik M., Kępa M., Wojtyczka R., Idzik D., Wąsik T. (2018). Phenolic Compounds Diminish Antibiotic Resistance of Staphylococcus Aureus Clinical Strains. Int. J. Environ. Res. Public Health.

[147] Khan R., Islam B., Akram M., Shakil S., Ahmad A.A., Ali S.M., Siddiqui M., Khan A. (2009). Antimicrobial Activity of Five Herbal Extracts Against Multi Drug Resistant (MDR) Strains of Bacteria and Fungus of Clinical Origin. Molecules.

[148] Marinaş I.C., Chifiriuc C., Oprea E., Lazăr V. (2014). Antimicrobial and Antioxidant Activities of Alcoholic Extracts Obtained from Vegetative Organs of A. Retroflexus. Roum. Arch. Microbiol. Immunol..

[149] Rangkadilok N., Tongchusak S., Boonhok R., Chaiyaroj S.C., Junyaprasert V.B., Buajeeb W., Akanimanee J., Raksasuk T., Suddhasthira T., Satayavivad J. (2012). In Vitro Antifungal Activities of Longan (Dimocarpus Longan Lour.) Seed Extract. Fitoterapia.

[150] Wang J., Zhang X., Gao L., Wang L., Song F., Zhang L., Wan Y. (2021). The Synergistic Antifungal Activity of Resveratrol with Azoles against *Candida albicans*. Lett. Appl. Microbiol..

[151] Xu M., Huang Z., Zhu W., Liu Y., Bai X., Zhang H. (2023). Fusarium-Derived Secondary Metabolites with Antimicrobial Effects. Molecules.

[152] Song X. (2024). Antibacterial, Antifungal, and Antiviral Bioactive Compounds from Natural Products. Molecules.

[153] Hu X., An S., Chu J., Liang B., Liao Y., Jiang J., Lin Y., Ye L., Liang H. (2023). Potential Inhibitors of Monkeypox Virus Revealed by Molecular Modeling Approach to Viral DNA Topoisomerase I. Molecules.

[154] Yu R., Li X., Yi P., Wen P., Wang S., Liao C., Song X., Wu H., He Z., Li C. (2023). Isolation and Identification of Chemical Compounds from Agaricus Blazei Murrill and Their In Vitro Antifungal Activities. Molecules.

[155] Abarova S., Alexova R., Dragomanova S., Solak A., Fagone P., Mangano K., Petralia M.C., Nicoletti F., Kalfin R., Tancheva L. (2024). Emerging Therapeutic Potential of Polyphenols from *Geranium sanguineum* L. in Viral Infections, Including SARS-CoV-2. Biomolecules.

[156] Abarova S., Tancheva L., Nikolov R., Serkedjieva J., Pavlova E., Bramanti A., Nicoletti F., Tzvetkov N.T. (2020). Preventive Effect of a Polyphenol-Rich Extract from *Geranium sanguineum* L. on Hepatic Drug Metabolism in Influenza Infected Mice. Sci. Pharm..

[157] Alexova R., Alexandrova S., Dragomanova S., Kalfin R., Solak A., Mehan S., Petralia M.C., Fagone P., Mangano K., Nicoletti F. (2023). Anti-COVID-19 Potential of Ellagic Acid and Polyphenols of *Punica granatum* L. Molecules.

[158] Álvarez-Martínez F.J., Rodríguez J.C., Borrás-Rocher F., Barrajón-Catalán E., Micol V. (2021). The Antimicrobial Capacity of Cistus Salviifolius and *Punica granatum* Plant Extracts against Clinical Pathogens Is Related to Their Polyphenolic Composition. Sci. Rep..

[159] Alam M.A. (2019). Anti-Hypertensive Effect of Cereal Antioxidant Ferulic Acid and Its Mechanism of Action. Front. Nutr..

[160] Jamee Shahwan A., Abed Y., Desormais I., Magne J., Preux P.M., Aboyans V., Lacroix P. (2019). Epidemiology of Coronary Artery Disease and Stroke and Associated Risk Factors in Gaza Community –Palestine. PLoS ONE.

[161] Li J., Liao R., Zhang S., Weng H., Liu Y., Tao T., Yu F., Li G., Wu J. (2023). Promising Remedies for Cardiovascular Disease: Natural Polyphenol Ellagic Acid and Its Metabolite Urolithins. Phytomedicine.

[162] Iqbal I., Wilairatana P., Saqib F., Nasir B., Wahid M., Latif M.F., Iqbal A., Naz R., Mubarak M.S. (2023). Plant Polyphenols and Their Potential Benefits on Cardiovascular Health: A Review. Molecules.

[163] Santhakumar A.B., Battino M., Alvarez-Suarez J.M. (2018). Dietary Polyphenols: Structures, Bioavailability and Protective Effects against Atherosclerosis. Food Chem. Toxicol..

[164] Malekmohammad K., Sewell R.D.E., Rafieian-Kopaei M. (2019). Antioxidants and Atherosclerosis: Mechanistic Aspects. Biomolecules.

[165] Sanches-Silva A., Testai L., Nabavi S.F., Battino M., Pandima Devi K., Tejada S., Sureda A., Xu S., Yousefi B., Majidinia M. (2020). Therapeutic Potential of Polyphenols in Cardiovascular Diseases: Regulation of mTOR Signaling Pathway. Pharmacol. Res..

[166] Haș I.M., Teleky B.-E., Vodnar D.-C., Ștefănescu B.E., Tit D.M., Nițescu M. (2023). Polyphenols and Cardiometabolic Health: Knowledge and Concern among Romanian People. Nutrients.

[167] Fuhrman B., Aviram M. (2001). Flavonoids Protect LDL from Oxidation and Attenuate Atherosclerosis. Curr. Opin. Lipidol..

[168] Banach M., Markuszewski L., Zasłonka J., Grzegorczyk J., Okoński P., Jegier B. (2004). The role of inflammation in the pathogenesis of atherosclerosis. Przegl. Epidemiol..

[169] Sirca T., Mureșan M., Pallag A., Marian E., Jurca T., Vicaș L., Tunduc I., Manole F., Ștefan L. (2024). The Role of Polyphenols in Modulating PON1 Activity Regarding Endothelial Dysfunction and Atherosclerosis. Int. J. Mol. Sci..

[170] Cimmino G., Muscoli S., De Rosa S., Cesaro A., Perrone M.A., Selvaggio S., Selvaggio G., Aimo A., Pedrinelli R., Mercuro G. (2023). Evolving Concepts in the Pathophysiology of Atherosclerosis: From Endothelial Dysfunction to Thrombus Formation through Multiple Shades of Inflammation. J. Cardiovasc. Med..

[171] Stein J.H., Keevil J.G., Wiebe D.A., Aeschlimann S., Folts J.D. (1999). Purple Grape Juice Improves Endothelial Function and Reduces the Susceptibility of LDL Cholesterol to Oxidation in Patients With Coronary Artery Disease. Circulation.

[172] Ciumărnean L., Milaciu M.V., Runcan O., Vesa S.C., Răchișan A.L., Negrean V., Perné M.-G., Donca V.I., Alexescu T.-G., Para I. (2020). The Effects of Flavonoids in Cardiovascular Diseases. Molecules.

[173] Rolnik A., Żuchowski J., Stochmal A., Olas B. (2020). Quercetin and Kaempferol Derivatives Isolated from Aerial Parts of Lens Culinaris Medik as Modulators of Blood Platelet Functions. Ind. Crops Prod..

[174] Ferrara L.A., Raimondi A.S., d’Episcopo L., Guida L., Dello Russo A., Marotta T. (2000). Olive Oil and Reduced Need for Antihypertensive Medications. Arch. Intern. Med..

[175] Yamagata K. (2019). Polyphenols Regulate Endothelial Functions and Reduce the Risk of Cardiovascular Disease. Curr. Pharm. Des..

[176] Elíes J., Cuíñas A., García-Morales V., Orallo F., Campos-Toimil M. (2011). *Trans*-resveratrol Simultaneously Increases Cytoplasmic Ca ^2+^ Levels and Nitric Oxide Release in Human Endothelial Cells. Mol. Nutr. Food Res..

[177] McKenna E., Smith J.S., Coll K.E., Mazack E.K., Mayer E.J., Antanavage J., Wiedmann R.T., Johnson R.G. (1996). Dissociation of Phospholamban Regulation of Cardiac Sarcoplasmic Reticulum Ca2+ATPase by Quercetin. J. Biol. Chem..

[178] Horie K., Nanashima N., Maeda H. (2019). Phytoestrogenic Effects of Blackcurrant Anthocyanins Increased Endothelial Nitric Oxide Synthase (eNOS) Expression in Human Endothelial Cells and Ovariectomized Rats. Molecules.

[179] Schewe T., Sadik C., Klotz L.-O., Yoshimoto T., Kühn H., Sies H. (2001). Polyphenols of Cocoa: Inhibition of Mammalian 15-Lipoxygenase. Biol. Chem..

[180] Kenny T.P., Keen C.L., Jones P., Kung H.-J., Schmitz H.H., Gershwin M.E. (2004). Pentameric Procyanidins Isolated from Theobroma Cacao Seeds Selectively Downregulate ErbB2 in Human Aortic Endothelial Cells. Exp. Biol. Med..

[181] Kenny T.P., Keen C.L., Jones P., Kung H.-J., Schmitz H.H., Gershwin M.E. (2004). Cocoa Procyanidins Inhibit Proliferation and Angiogenic Signals in Human Dermal Microvascular Endothelial Cells Following Stimulation by Low-Level H_2_O_2_. Exp. Biol. Med..

[182] Kelishadi R. (2005). Cacao to cocoa to chocolate: Healthy food?. ARYA J..

[183] Choy K.W., Murugan D., Leong X.-F., Abas R., Alias A., Mustafa M.R. (2019). Flavonoids as Natural Anti-Inflammatory Agents Targeting Nuclear Factor-Kappa B (NFκB) Signaling in Cardiovascular Diseases: A Mini Review. Front. Pharmacol..

[184] Dias M.C., Pinto D.C.G.A., Silva A.M.S. (2021). Plant Flavonoids: Chemical Characteristics and Biological Activity. Molecules.

[185] Liao H., Ye J., Gao L., Liu Y. (2021). The Main Bioactive Compounds of Scutellaria Baicalensis Georgi. for Alleviation of Inflammatory Cytokines: A Comprehensive Review. Biomed. Pharmacother..

[186] Al-Khayri J.M., Sahana G.R., Nagella P., Joseph B.V., Alessa F.M., Al-Mssallem M.Q. (2022). Flavonoids as Potential Anti-Inflammatory Molecules: A Review. Molecules.

[187] Sychrová A., Škovranová G., Čulenová M., Bittner Fialová S. (2022). Prenylated Flavonoids in Topical Infections and Wound Healing. Molecules.

[188] Krauth V., Bruno F., Pace S., Jordan P.M., Temml V., Preziosa Romano M., Khan H., Schuster D., Rossi A., Filosa R. (2023). Highly Potent and Selective 5-Lipoxygenase Inhibition by New, Simple Heteroaryl-Substituted Catechols for Treatment of Inflammation. Biochem. Pharmacol..

[189] Martinez J., Moreno J.J. (2000). Effect of Resveratrol, a Natural Polyphenolic Compound, on Reactive Oxygen Species and Prostaglandin Production. Biochem. Pharmacol..

[190] Pey A.L., Megarity C.F., Timson D.J. (2019). NAD(P)H Quinone Oxidoreductase (NQO1): An Enzyme Which Needs Just Enough Mobility, in Just the Right Places. Biosci. Rep..

[191] Wyss-Coray T. (2016). Ageing, Neurodegeneration and Brain Rejuvenation. Nature.

[192] Lee B.K., Hyun S.-W., Jung Y.-S. (2020). Yuzu and Hesperidin Ameliorate Blood-Brain Barrier Disruption during Hypoxia via Antioxidant Activity. Antioxidants.

[193] Nájera-Maldonado J.M., Salazar R., Alvarez-Fitz P., Acevedo-Quiroz M., Flores-Alfaro E., Hernández-Sotelo D., Espinoza-Rojo M., Ramírez M. (2024). Phenolic Compounds of Therapeutic Interest in Neuroprotection. JoX.

[194] Tian Y., Meng L., Zhang Z. (2020). What Is Strain in Neurodegenerative Diseases?. Cell. Mol. Life Sci..

[195] Ranilla L.G., Kwon Y.-I., Apostolidis E., Shetty K. (2010). Phenolic Compounds, Antioxidant Activity and in Vitro Inhibitory Potential against Key Enzymes Relevant for Hyperglycemia and Hypertension of Commonly Used Medicinal Plants, Herbs and Spices in Latin America. Bioresour. Technol..

[196] Lopes G., Gomes E., Barbosa M., Bernardo J., Valentão P. (2022). Camel Grass Phenolic Compounds: Targeting Inflammation and Neurologically Related Conditions. Molecules.

[197] Arias-Sánchez R.A., Torner L., Fenton Navarro B. (2023). Polyphenols and Neurodegenerative Diseases: Potential Effects and Mechanisms of Neuroprotection. Molecules.

[198] Montes F.O., Váquez-Hernádez A., Fenton-Navarro B. (2019). Active Compounds of Medicinal Plants, Mechanism for Antioxidant and Beneficial Effects. Phyton.

[199] Di Meo F., Valentino A., Petillo O., Peluso G., Filosa S., Crispi S. (2020). Bioactive Polyphenols and Neuromodulation: Molecular Mechanisms in Neurodegeneration. Int. J. Mol. Sci..

[200] Kasprzak-Drozd K., Oniszczuk T., Stasiak M., Oniszczuk A. (2021). Beneficial Effects of Phenolic Compounds on Gut Microbiota and Metabolic Syndrome. Int. J. Mol. Sci..

[201] Figueira I., Menezes R., Macedo D., Costa I., Dos Santos C.N. (2017). Polyphenols Beyond Barriers: A Glimpse into the Brain. Curr. Neuropharmacol..

[202] Lamport D.J., Williams C.M. (2021). Polyphenols and Cognition In Humans: An Overview of Current Evidence from Recent Systematic Reviews and Meta-Analyses. Brain Plast..

[203] Bari A., Shah S.M.M., Al-Joufi F.A., Shah S.W.A., Shoaib M., Shah I., Zahoor M., Ahmed M.N., Ghias M., Shah S.M.H. (2022). Effects of Artemisia Macrocephala Jacquem on Memory Deficits and Brain Oxidative Stress in Streptozotocin-Induced Diabetic Mice. Molecules.

[204] Rojas-García A., Fernández-Ochoa Á., Cádiz-Gurrea M.D.L.L., Arráez-Román D., Segura-Carretero A. (2023). Neuroprotective Effects of Agri-Food By-Products Rich in Phenolic Compounds. Nutrients.

[205] Murillo Ortíz B., Ramírez Emiliano J., Ramos-Rodríguez E., Martínez-Garza S., Macías-Cervantes H., Solorio-Meza S., Pereyra-Nobara T.A. (2016). Brain-Derived Neurotrophic Factor Plasma Levels and Premature Cognitive Impairment/Dementia in Type 2 Diabetes. World J. Diabetes.

[206] Bathina S., Das U.N. (2015). Brain-Derived Neurotrophic Factor and Its Clinical Implications. Arch. Med. Sci..

[207] Zeng P., Fang M., Zhao H., Guo J. (2021). A Network Pharmacology Approach to Uncover the Key Ingredients in Ginkgo Folium and Their Anti-Alzheimer’s Disease Mechanisms. Aging.

[208] Zhang Y., Yu W., Zhang L., Wang M., Chang W. (2022). The Interaction of Polyphenols and the Gut Microbiota in Neurodegenerative Diseases. Nutrients.

[209] Campos-Esparza M.R., Sánchez-Gómez M.V., Matute C. (2009). Molecular Mechanisms of Neuroprotection by Two Natural Antioxidant Polyphenols. Cell Calcium.

[210] Carecho R., Figueira I., Terrasso A.P., Godinho-Pereira J., De Oliveira Sequeira C., Pereira S.A., Milenkovic D., Leist M., Brito C., Nunes Dos Santos C. (2022). Circulating (Poly)Phenol Metabolites: Neuroprotection in a 3D Cell Model of Parkinson’s Disease. Mol. Nutr. Food Res..

[211] Chesworth R., Gamage R., Ullah F., Sonego S., Millington C., Fernandez A., Liang H., Karl T., Münch G., Niedermayer G. (2021). Spatial Memory and Microglia Activation in a Mouse Model of Chronic Neuroinflammation and the Anti-Inflammatory Effects of Apigenin. Front. Neurosci..

[212] Saxena P., Selvaraj K., Khare S.K., Chaudhary N. (2022). Superoxide Dismutase as Multipotent Therapeutic Antioxidant Enzyme: Role in Human Diseases. Biotechnol. Lett..

[213] Foti M.C. (2010). Antioxidant Properties of Phenols. J. Pharm. Pharmacol..

[214] Chico L., Ienco E.C., Bisordi C., Lo Gerfo A., Petrozzi L., Petrucci A., Mancuso M., Siciliano G. (2018). Amyotrophic Lateral Sclerosis and Oxidative Stress: A Double-Blind Therapeutic Trial After Curcumin Supplementation. CNS Neurol. Disord. Drug Targets.

[215] Dufour C., Dangles O. (2005). Flavonoid–Serum Albumin Complexation: Determination of Binding Constants and Binding Sites by Fluorescence Spectroscopy. Biochim. Biophys. Acta (BBA) Gen. Subj..

[216] Kitson T.M. (2004). Spectrophotometric and Kinetic Studies on the Binding of the Bioflavonoid Quercetin to Bovine Serum Albumin. Biosci. Biotechnol. Biochem..

[217] Kesse-Guyot E., Fezeu L., Andreeva V.A., Touvier M., Scalbert A., Hercberg S., Galan P. (2012). Total and Specific Polyphenol Intakes in Midlife Are Associated with Cognitive Function Measured 13 Years Later. J. Nutr..

[218] Wang L., Cao D., Wu H., Jia H., Yang C., Zhang L. (2019). Fisetin Prolongs Therapy Window of Brain Ischemic Stroke Using Tissue Plasminogen Activator: A Double-Blind Randomized Placebo-Controlled Clinical Trial. Clin. Appl. Thromb. Hemost..

[219] Moussa C., Hebron M., Huang X., Ahn J., Rissman R.A., Aisen P.S., Turner R.S. (2017). Resveratrol Regulates Neuro-Inflammation and Induces Adaptive Immunity in Alzheimer’s Disease. J. Neuroinflamm..

[220] Morris G., Gamage E., Travica N., Berk M., Jacka F.N., O’Neil A., Puri B.K., Carvalho A.F., Bortolasci C.C., Walder K. (2021). Polyphenols as Adjunctive Treatments in Psychiatric and Neurodegenerative Disorders: Efficacy, Mechanisms of Action, and Factors Influencing Inter-Individual Response. Free Radic. Biol. Med..

[221] Micek A., Owczarek M., Jurek J., Guerrera I., Torrisi S.A., Grosso G., Alshatwi A.A., Godos J. (2022). Anthocyanin-Rich Fruits and Mental Health Outcomes in an Italian Cohort. J. Berry Res..

[222] Kumar V., Singh D.D., Lakhawat S.S., Yasmeen N., Pandey A., Singla R.K. (2022). Biogenic Phytochemicals Modulating Obesity: From Molecular Mechanism to Preventive and Therapeutic Approaches. Evid.-Based Complement. Altern. Med..

[223] Mamun M.A.A., Rakib A., Mandal M., Kumar S., Singla B., Singh U.P. (2024). Polyphenols: Role in Modulating Immune Function and Obesity. Biomolecules.

[224] Herrera-Martínez A.D., Herrero-Aguayo V., Pérez-Gómez J.M., Gahete M.D., Luque R.M. (2022). Inflammasomes: Cause or Consequence of Obesity-associated Comorbidities in Humans. Obesity.

[225] Liu J., Wang H., Zeng D., Xiong J., Luo J., Chen X., Chen T., Xi Q., Sun J., Ren X. (2023). The Novel Importance of miR-143 in Obesity Regulation. Int. J. Obes..

[226] Zatterale F., Longo M., Naderi J., Raciti G.A., Desiderio A., Miele C., Beguinot F. (2020). Chronic Adipose Tissue Inflammation Linking Obesity to Insulin Resistance and Type 2 Diabetes. Front. Physiol..

[227] Timmers S., Konings E., Bilet L., Houtkooper R.H., van de Weijer T., Goossens G.H., Hoeks J., van der Krieken S., Ryu D., Kersten S. (2011). Calorie Restriction-like Effects of 30 Days of Resveratrol Supplementation on Energy Metabolism and Metabolic Profile in Obese Humans. Cell Metab..

[228] Jimenez-Gomez Y., Mattison J.A., Pearson K.J., Martin-Montalvo A., Palacios H.H., Sossong A.M., Ward T.M., Younts C.M., Lewis K., Allard J.S. (2013). Resveratrol Improves Adipose Insulin Signaling and Reduces the Inflammatory Response in Adipose Tissue of Rhesus Monkeys on High-Fat, High-Sugar Diet. Cell Metab..

[229] Incalza M.A., D’Oria R., Natalicchio A., Perrini S., Laviola L., Giorgino F. (2018). Oxidative Stress and Reactive Oxygen Species in Endothelial Dysfunction Associated with Cardiovascular and Metabolic Diseases. Vasc. Pharmacol..

[230] Kardum N., Glibetic M., Toldrá F. (2018). Chapter Three—Polyphenols and Their Interactions With Other Dietary Compounds: Implications for Human Health. Advances in Food and Nutrition Research.

[231] Aghababaei F., Hadidi M. (2023). Recent Advances in Potential Health Benefits of Quercetin. Pharmaceuticals.

[232] Sahebkar A. (2014). Are Curcuminoids Effective C-Reactive Protein-Lowering Agents in Clinical Practice? Evidence from a Meta-Analysis: Curcuminoids and crp. Phytother. Res..

[233] Ji X., Shi S., Liu B., Shan M., Tang D., Zhang W., Zhang Y., Zhang L., Zhang H., Lu C. (2019). Bioactive Compounds from Herbal Medicines to Manage Dyslipidemia. Biomed. Pharmacother..

[234] Hachimura S., Totsuka M., Hosono A. (2018). Immunomodulation by Food: Impact on Gut Immunity and Immune Cell Function. Biosci. Biotechnol. Biochem..

[235] Dugo L., Belluomo M.G., Fanali C., Russo M., Cacciola F., Maccarrone M., Sardanelli A.M. (2017). Effect of Cocoa Polyphenolic Extract on Macrophage Polarization from Proinflammatory M1 to Anti-Inflammatory M2 State. Oxidative Med. Cell. Longev..

[236] Ben Lagha A., Azelmat J., Vaillancourt K., Grenier D. (2021). A Polyphenolic Cinnamon Fraction Exhibits Anti-Inflammatory Properties in a Monocyte/Macrophage Model. PLoS ONE.

[237] Song Y., Jung Y.S., Park S., Park H.S., Lee S.J., Maeng S., Kim H., Kim D., Park K.W., Kang H. (2023). Anti-Inflammatory Effects and Macrophage Activation Induced by Bioavailable Cinnamon Polyphenols in Mice. Mol. Nutr. Food Res..

[238] Vivier E., Tomasello E., Baratin M., Walzer T., Ugolini S. (2008). Functions of Natural Killer Cells. Nat. Immunol..

[239] Lu H.-Y., Peng T.-S., Hu X.-D., Li S.-J., Luo M., He Y.-H., Nie T. (2015). Quercetin Potentiates the Effect of Γδ T Cells via Modulating the Expressions of Granzyme B, Perforin and IFN-γ and Also Regulates the Wnt/β-Catenin Signalling Pathway in Human Colon Cancer Cells. Bangladesh J. Pharmacol..

[240] Burkard M., Leischner C., Lauer U.M., Busch C., Venturelli S., Frank J. (2017). Dietary Flavonoids and Modulation of Natural Killer Cells: Implications in Malignant and Viral Diseases. J. Nutr. Biochem..

[241] Lee Y., Shin H., Kim J. (2021). In Vivo Anti-Cancer Effects of Resveratrol Mediated by NK Cell Activation. J. Innate Immun..

[242] Venturelli S., Berger A., Böcker A., Busch C., Weiland T., Noor S., Leischner C., Schleicher S., Mayer M., Weiss T.S. (2013). Resveratrol as a Pan-HDAC Inhibitor Alters the Acetylation Status of Histone [Corrected] Proteins in Human-Derived Hepatoblastoma Cells. PLoS ONE.

[243] Bae J.-H., Kim J.-Y., Kim M.-J., Chang S.-H., Park Y.-S., Son C.-H., Park S.-J., Chung J.-S., Lee E.-Y., Kim S.-H. (2010). Quercetin Enhances Susceptibility to NK Cell-Mediated Lysis of Tumor Cells through Induction of NKG2D Ligands and Suppression of HSP70. J. Immunother..

[244] Hasima N., Ozpolat B. (2014). Regulation of Autophagy by Polyphenolic Compounds as a Potential Therapeutic Strategy for Cancer. Cell Death Dis..

[245] Kiran S., Kumar V., Murphy E.A., Enos R.T., Singh U.P. (2021). High Fat Diet-Induced CD8+ T Cells in Adipose Tissue Mediate Macrophages to Sustain Low-Grade Chronic Inflammation. Front. Immunol..

[246] Mohammadi A., Blesso C.N., Barreto G.E., Banach M., Majeed M., Sahebkar A. (2019). Macrophage Plasticity, Polarization and Function in Response to Curcumin, a Diet-Derived Polyphenol, as an Immunomodulatory Agent. J. Nutr. Biochem..

[247] Islam T., Koboziev I., Albracht-Schulte K., Mistretta B., Scoggin S., Yosofvand M., Moussa H., Zabet-Moghaddam M., Ramalingam L., Gunaratne P.H. (2021). Curcumin Reduces Adipose Tissue Inflammation and Alters Gut Microbiota in Diet-Induced Obese Male Mice. Mol. Nutr. Food Res..

[248] Çetinalp P., Değirmencioğlu S., Küçük S.T., Seyithanoğlu M., İyidoğan Y.Ö., Koçak H. (2022). Association of Different Doses of Curcumin with Preadipocyte-Adipocyte Differentiation and Inflammatory Statu. https://www.researchsquare.com/article/rs-1974683/v1.

[249] Kang L., Heng W., Yuan A., Baolin L., Fang H. (2010). Resveratrol Modulates Adipokine Expression and Improves Insulin Sensitivity in Adipocytes: Relative to Inhibition of Inflammatory Responses. Biochimie.

[250] Wang B., Sun J., Li L., Zheng J., Shi Y., Le G. (2014). Regulatory Effects of Resveratrol on Glucose Metabolism and T-Lymphocyte Subsets in the Development of High-Fat Diet-Induced Obesity in C57BL/6 Mice. Food Funct..

[251] Kang J.-H., Kim C.-S., Han I.-S., Kawada T., Yu R. (2007). Capsaicin, a Spicy Component of Hot Peppers, Modulates Adipokine Gene Expression and Protein Release from Obese-Mouse Adipose Tissues and Isolated Adipocytes, and Suppresses the Inflammatory Responses of Adipose Tissue Macrophages. FEBS Lett..

[252] Collins B., Hoffman J., Martinez K., Grace M., Lila M.A., Cockrell C., Nadimpalli A., Chang E., Chuang C.-C., Zhong W. (2016). A Polyphenol-Rich Fraction Obtained from Table Grapes Decreases Adiposity, Insulin Resistance and Markers of Inflammation and Impacts Gut Microbiota in High-Fat-Fed Mice. J. Nutr. Biochem..

[253] Chuang C.-C., Shen W., Chen H., Xie G., Jia W., Chung S., McIntosh M.K. (2012). Differential Effects of Grape Powder and Its Extract on Glucose Tolerance and Chronic Inflammation in High-Fat-Fed Obese Mice. J. Agric. Food Chem..

[254] Rahman S.U., Huang Y., Zhu L., Chu X., Junejo S.A., Zhang Y., Khan I.M., Li Y., Feng S., Wu J. (2020). Tea Polyphenols Attenuate Liver Inflammation by Modulating Obesity-Related Genes and down-Regulating COX-2 and iNOS Expression in High Fat-Fed Dogs. BMC Vet. Res..

[255] Ferraz C.R., Carvalho T.T., Manchope M.F., Artero N.A., Rasquel-Oliveira F.S., Fattori V., Casagrande R., Verri W.A. (2020). Therapeutic Potential of Flavonoids in Pain and Inflammation: Mechanisms of Action, Pre-Clinical and Clinical Data, and Pharmaceutical Development. Molecules.

[256] Solnier J., Chang C., Pizzorno J. (2023). Consideration for Flavonoid-Containing Dietary Supplements to Tackle Deficiency and Optimize Health. Int. J. Mol. Sci..

[257] Garbetta A., Nicassio L., D’Antuono I., Cardinali A., Linsalata V., Attolico G., Minervini F. (2018). Influence of in Vitro Digestion Process on Polyphenolic Profile of Skin Grape (Cv. Italia) and on Antioxidant Activity in Basal or Stressed Conditions of Human Intestinal Cell Line (HT-29). Food Res. Int..

[258] Graziani G., D’Argenio G., Tuccillo C., Loguercio C., Ritieni A., Morisco F., Del Vecchio Blanco C., Fogliano V., Romano M. (2005). Apple Polyphenol Extracts Prevent Damage to Human Gastric Epithelial Cells in Vitro and to Rat Gastric Mucosa in Vivo. Gut.

[259] Schaefer S., Baum M., Eisenbrand G., Dietrich H., Will F., Janzowski C. (2006). Polyphenolic Apple Juice Extracts and Their Major Constituents Reduce Oxidative Damage in Human Colon Cell Lines. Mol. Nutr. Food Res..

[260] Seeram N.P., Adams L.S., Henning S.M., Niu Y., Zhang Y., Nair M.G., Heber D. (2005). In Vitro Antiproliferative, Apoptotic and Antioxidant Activities of Punicalagin, Ellagic Acid and a Total Pomegranate Tannin Extract Are Enhanced in Combination with Other Polyphenols as Found in Pomegranate Juice. J. Nutr. Biochem..

[261] Nunes C., Ferreira E., Freitas V., Almeida L., Barbosa R.M., Laranjinha J. (2013). Intestinal Anti-Inflammatory Activity of Red Wine Extract: Unveiling the Mechanisms in Colonic Epithelial Cells. Food Funct..

[262] Gessner D.K., Ringseis R., Siebers M., Keller J., Kloster J., Wen G., Eder K. (2012). Inhibition of the Pro-Inflammatory NF-κB Pathway by a Grape Seed and Grape Marc Meal Extract in Intestinal Epithelial Cells. J. Anim. Physiol. Anim. Nutr..

[263] Oz H.S., Chen T.S., McClain C.J., de Villiers W.J.S. (2005). Antioxidants as Novel Therapy in a Murine Model of Colitis. J. Nutr. Biochem..

[264] Brückner M., Westphal S., Domschke W., Kucharzik T., Lügering A. (2012). Green Tea Polyphenol Epigallocatechin-3-Gallate Shows Therapeutic Antioxidative Effects in a Murine Model of Colitis. J. Crohns Colitis.

[265] Jiao X., Wang Y., Lin Y., Lang Y., Li E., Zhang X., Zhang Q., Feng Y., Meng X., Li B. (2019). Blueberry Polyphenols Extract as a Potential Prebiotic with Anti-Obesity Effects on C57BL/6 J Mice by Modulating the Gut Microbiota. J. Nutr. Biochem..

[266] Soeng S., Evacuasiany E., Widowati W., Fauziah N., Manik V., Maesaroh M. (2015). Inhibitory Potential of Rambutan Seeds Extract and Fractions on Adipogenesis in 3T3-L1 Cell Line. J. Exp. Integr. Med..

[267] Pinent M., Bladé M.C., Salvadó M.J., Arola L., Hackl H., Quackenbush J., Trajanoski Z., Ardévol A. (2005). Grape-Seed Derived Procyanidins Interfere with Adipogenesis of 3T3-L1 Cells at the Onset of Differentiation. Int. J. Obes..

[268] Zhou F., Guo J., Han X., Gao Y., Chen Q., Huang W., Zhan J., Huang D., You Y. (2020). Cranberry Polyphenolic Extract Exhibits an Antiobesity Effect on High-Fat Diet-Fed Mice through Increased Thermogenesis. J. Nutr..

[269] Van der Zande H.J.P., Lambooij J.M., Chavanelle V., Zawistowska-Deniziak A., Otero Y., Otto F., Lantier L., McGuinness O.P., Le Joubioux F., Giera M. (2021). Effects of a Novel Polyphenol-Rich Plant Extract on Body Composition, Inflammation, Insulin Sensitivity, and Glucose Homeostasis in Obese Mice. Int. J. Obes..

[270] Abdul Satar N., Ismail M.N., Yahaya B.H. (2021). Synergistic Roles of Curcumin in Sensitising the Cisplatin Effect on a Cancer Stem Cell-Like Population Derived from Non-Small Cell Lung Cancer Cell Lines. Molecules.

[271] He Y.-Z., Yu S.-L., Li X.-N., Bai X.-H., Li H.-T., Liu Y.-C., Lv B.-L., Zhao X.-M., Wei D., Zhang H.-L. (2022). Curcumin Increases Crizotinib Sensitivity through the Inactivation of Autophagy via Epigenetic Modulation of the miR-142-5p/Ulk1 Axis in Non-Small Cell Lung Cancer. Cancer Biomark..

[272] Zhu C., Fang Z., Peng L., Gao F., Peng W., Song F. (2022). Curcumin Suppresses the Progression of Colorectal Cancer by Improving Immunogenic Cell Death Caused by Irinotecan. Chemotherapy.

[273] Yin J., Wang L., Wang Y., Shen H., Wang X., Wu L. (2019). Curcumin Reverses Oxaliplatin Resistance in Human Colorectal Cancer via Regulation of TGF-β/Smad2/3 Signaling Pathway. Onco Targets Ther..

[274] Xu T., Guo P., Pi C., He Y., Yang H., Hou Y., Feng X., Jiang Q., Wei Y., Zhao L. (2020). Synergistic Effects of Curcumin and 5-Fluorouracil on the Hepatocellular Carcinoma In Vivo and Vitro through Regulating the Expression of COX-2 and NF-κB. J. Cancer.

[275] Bahman A.A., Abaza M.S.I., Khoushiash S.I., Al-Attiyah R.J. (2018). Sequence-dependent Effect of Sorafenib in Combination with Natural Phenolic Compounds on Hepatic Cancer Cells and the Possible Mechanism of Action. Int. J. Mol. Med..

[276] Li S., Yuan S., Zhao Q., Wang B., Wang X., Li K. (2018). Quercetin Enhances Chemotherapeutic Effect of Doxorubicin against Human Breast Cancer Cells While Reducing Toxic Side Effects of It. Biomed. Pharmacother..

[277] Ozkan E., Bakar-Ates F. (2020). Potentiation of the Effect of Lonidamine by Quercetin in MCF-7 Human Breast Cancer Cells through Downregulation of MMP-2/9 mRNA Expression. Acad. Bras. Cienc..

[278] Hyun H.B., Moon J.Y., Cho S.K. (2018). Quercetin Suppresses CYR61-Mediated Multidrug Resistance in Human Gastric Adenocarcinoma AGS Cells. Molecules.

[279] Chen M., Duan C., Pan J. (2021). Quercetin Increases Doxorubicin-Induced Apoptosis Through Oxidative DNA Damage in KATO III Gastric Cancer Cells. Iran. Red Crescent Med. J..

[280] Zhou Y., Zhang J., Wang K., Han W., Wang X., Gao M., Wang Z., Sun Y., Yan H., Zhang H. (2020). Quercetin Overcomes Colon Cancer Cells Resistance to Chemotherapy by Inhibiting Solute Carrier Family 1, Member 5 Transporter. Eur. J. Pharmacol..

[281] Erdoğan M.K., Ağca C.A., Aşkın H. (2022). Quercetin and Luteolin Improve the Anticancer Effects of 5-Fluorouracil in Human Colorectal Adenocarcinoma In Vitro Model: A Mechanistic Insight. Nutr. Cancer.

[282] Zhang P., Zhang J., Zhao L., Li S., Li K. (2022). Quercetin Attenuates the Cardiotoxicity of Doxorubicin–Cyclophosphamide Regimen and Potentiates Its Chemotherapeutic Effect against Triple-negative Breast Cancer. Phytother. Res..

[283] Yang M.-D., Sun Y., Zhou W.-J., Xie X.-Z., Zhou Q.-M., Lu Y.-Y., Su S.-B. (2021). Resveratrol Enhances Inhibition Effects of Cisplatin on Cell Migration and Invasion and Tumor Growth in Breast Cancer MDA-MB-231 Cell Models In Vivo and In Vitro. Molecules.

[284] Zhang W., Jiang H., Chen Y., Ren F. (2019). Resveratrol Chemosensitizes Adriamycin-Resistant Breast Cancer Cells by Modulating miR-122-5p. J. Cell Biochem..

[285] Qin S.-H., Lau A.T.Y., Liang Z.-L., Tan H.W., Ji Y.-C., Zhong Q.-H., Zhao X.-Y., Xu Y.-M. (2020). Resveratrol Promotes Tumor Microvessel Growth via Endoglin and Extracellular Signal-Regulated Kinase Signaling Pathway and Enhances the Anticancer Efficacy of Gemcitabine against Lung Cancer. Cancers.

[286] Cipolletti M., Montalesi E., Nuzzo M.T., Fiocchetti M., Ascenzi P., Marino M. (2019). Potentiation of Paclitaxel Effect by Resveratrol in Human Breast Cancer Cells by Counteracting the 17β-Estradiol/Estrogen Receptor α/Neuroglobin Pathway. J. Cell Physiol..

[287] Rizza S., Muniyappa R., Iantorno M., Kim J., Chen H., Pullikotil P., Senese N., Tesauro M., Lauro D., Cardillo C. (2011). Citrus Polyphenol Hesperidin Stimulates Production of Nitric Oxide in Endothelial Cells While Improving Endothelial Function and Reducing Inflammatory Markers in Patients with Metabolic Syndrome. J. Clin. Endocrinol. Metab..

[288] Cospite M. (1994). Double-Blind, Placebo-Controlled Evaluation of Clinical Activity and Safety of Daflon 500 Mg in the Treatment of Acute Hemorrhoids. Angiology.

[289] Bogdanski P., Suliburska J., Szulinska M., Stepien M., Pupek-Musialik D., Jablecka A. (2012). Green Tea Extract Reduces Blood Pressure, Inflammatory Biomarkers, and Oxidative Stress and Improves Parameters Associated with Insulin Resistance in Obese, Hypertensive Patients. Nutr. Res..

[290] Javadi F., Ahmadzadeh A., Eghtesadi S., Aryaeian N., Zabihiyeganeh M., Rahimi Foroushani A., Jazayeri S. (2017). The Effect of Quercetin on Inflammatory Factors and Clinical Symptoms in Women with Rheumatoid Arthritis: A Double-Blind, Randomized Controlled Trial. J. Am. Coll. Nutr..

[291] Gallelli G., Cione E., Serra R., Leo A., Citraro R., Matricardi P., Di Meo C., Bisceglia F., Caroleo M.C., Basile S. (2020). Nano-hydrogel Embedded with Quercetin and Oleic Acid as a New Formulation in the Treatment of Diabetic Foot Ulcer: A Pilot Study. Int. Wound J..

[292] Zargaran A., Borhani-Haghighi A., Salehi-Marzijarani M., Faridi P., Daneshamouz S., Azadi A., Sadeghpour H., Sakhteman A., Mohagheghzadeh A. (2018). Evaluation of the Effect of Topical Chamomile (*Matricaria chamomilla* L.) Oleogel as Pain Relief in Migraine without Aura: A Randomized, Double-Blind, Placebo-Controlled, Crossover Study. Neurol. Sci..

[293] Shavandi M., Moini A., Shakiba Y., Mashkorinia A., Dehghani M., Asar S., Kiani A. (2017). Silymarin (Livergol®) Decreases Disease Activity Score in Patients with Rheumatoid Arthritis: A Non-Randomized Single-Arm Clinical Trial. Iran. J. Allergy Asthma Immunol..

[294] Feragalli B., Dugall M., Luzzi R., Ledda A., Hosoi M., Belcaro G., Cesarone M.R. (2019). Pycnogenol®: Supplementary Management of Symptomatic Osteoarthritis with a Patch. An Observational Registry Study. Minerva Endocrinol..

[295] Cisár P., Jány R., Waczulíková I., Sumegová K., Muchová J., Vojtassák J., Duraćková Z., Lisý M., Rohdewald P. (2008). Effect of Pine Bark Extract (Pycnogenol) on Symptoms of Knee Osteoarthritis. Phytother. Res..

[296] Farid R., Mirfeizi Z., Mirheidari M., Rezaieyazdi Z., Mansouri H., Esmaelli H., Zibadi S., Rohdewald P., Watson R.R. (2007). Pycnogenol Supplementation Reduces Pain and Stiffness and Improves Physical Function in Adults with Knee Osteoarthritis. Nutr. Res..

[297] Awan F.T., Jones J.A., Maddocks K., Poi M., Grever M.R., Johnson A., Byrd J.C., Andritsos L.A. (2016). A Phase 1 Clinical Trial of Flavopiridol Consolidation in Chronic Lymphocytic Leukemia Patients Following Chemoimmunotherapy. Ann. Hematol..

[298] Amato B., Compagna R., Amato M., Gallelli L., de Franciscis S., Serra R. (2015). Aterofisiol(®) in Carotid Plaque Evolution. Drug Des. Devel Ther..

[299] Biesinger S., Michaels H.A., Quadros A.S., Qian Y., Rabovsky A.B., Badger R.S., Jalili T. (2016). A Combination of Isolated Phytochemicals and Botanical Extracts Lowers Diastolic Blood Pressure in a Randomized Controlled Trial of Hypertensive Subjects. Eur. J. Clin. Nutr..

[300] Bhatt J.K., Thomas S., Nanjan M.J. (2012). Resveratrol Supplementation Improves Glycemic Control in Type 2 Diabetes Mellitus. Nutr. Res..

[301] Martínez De Toda I., González-Sánchez M., Díaz-Del Cerro E., Valera G., Carracedo J., Guerra-Pérez N. (2023). Sex Differences in Markers of Oxidation and Inflammation. Implications for Ageing. Mech. Ageing Dev..

[302] Duda-Chodak A., Tarko T. (2023). Possible Side Effects of Polyphenols and Their Interactions with Medicines. Molecules.

[303] Fukami T., Yokoi T., Nakajima M. (2022). Non-P450 Drug-Metabolizing Enzymes: Contribution to Drug Disposition, Toxicity, and Development. Annu. Rev. Pharmacol. Toxicol..

[304] Esteves F., Rueff J., Kranendonk M. (2021). The Central Role of Cytochrome P450 in Xenobiotic Metabolism—A Brief Review on a Fascinating Enzyme Family. J. Xenobiotics.

[305] Bhamre Vaibhav G., Deore Pranjal D., Amrutkar Rakesh D., Patil Vinod R. (2022). Polyphenols: The Interactions with CYP Isoenzymes and Effect on Pharmacokinetics of Drugs. Curr. Trends Pharm. Pharm. Chem..

